# Development of a novel disulfidptosis-related lncRNA signature for prognostic and immune response prediction in clear cell renal cell carcinoma

**DOI:** 10.1038/s41598-024-51197-2

**Published:** 2024-01-05

**Authors:** Ning Wang, Yifeng Hu, Shasha Wang, Qin Xu, Xiaojing Jiao, Yanliang Wang, Lei Yan, Huixia Cao, Fengmin Shao

**Affiliations:** 1 Henan Provincial Key Laboratory of Kidney Disease and Immunology, Henan Provincial Clinical Research Center for Kidney Disease, Department of Nephrology, Henan Provincial People’s Hospital, People’s Hospital of Zhengzhou University, Zhengzhou, 450003 China; 2 Academy of Medical Sciences, Zhengzhou University, Zhengzhou, 450052 China

**Keywords:** Renal cell carcinoma, Renal cell carcinoma, Tumour biomarkers, Computational models

## Abstract

Disulfidptosis, a novel form of regulated cell death, occurs due to the aberrant accumulation of intracellular cystine and other disulfides. Moreover, targeting disulfidptosis could identify promising approaches for cancer treatment. Long non-coding RNAs (lncRNAs) are known to be critically implicated in clear cell renal cell carcinoma (ccRCC) development. Currently, the involvement of disulfidptosis-related lncRNAs in ccRCC is yet to be elucidated. This study primarily dealt with identifying and validating a disulfidptosis-related lncRNAs-based signature for predicting the prognosis and immune landscape of individuals with ccRCC. Clinical and RNA sequencing data of ccRCC samples were accessed from The Cancer Genome Atlas (TCGA) database. Pearson correlation analysis was conducted for the identification of the disulfidptosis-related lncRNAs. Additionally, univariate Cox regression analysis, Least Absolute Shrinkage and Selection Operator Cox regression, and stepwise multivariate Cox analysis were executed to develop a novel risk prognostic model. The prognosis-predictive capacity of the model was then assessed using an integrated method. Variation in biological function was noted using GO, KEGG, and GSEA. Additionally, immune cell infiltration, the tumor mutational burden (TMB), and tumor immune dysfunction and exclusion (TIDE) scores were calculated to investigate differences in the immune landscape. Finally, the expression of hub disulfidptosis-related lncRNAs was validated using qPCR. We established a novel signature comprised of eight lncRNAs that were associated with disulfidptosis (SPINT1-AS1, AL121944.1, AC131009.3, AC104088.3, AL035071.1, LINC00886, AL035587.2, and AC007743.1). Kaplan–Meier and receiver operating characteristic curves demonstrated the acceptable predictive potency of the model. The nomogram and C-index confirmed the strong correlation between the risk signature and clinical decision-making. Furthermore, immune cell infiltration analysis and ssGSEA revealed significantly different immune statuses among risk groups. TMB analysis revealed the link between the high-risk group and high TMB. It is worth noting that the cumulative effect of the patients belonging to the high-risk group and having elevated TMB led to decreased patient survival times. The high-risk group depicted greater TIDE scores in contrast with the low-risk group, indicating greater potential for immune escape. Finally, qPCR validated the hub disulfidptosis-related lncRNAs in cell lines. The established novel signature holds potential regarding the prognosis prediction of individuals with ccRCC as well as predicting their responses to immunotherapy.

## Introduction

Clear cell renal cell carcinoma (ccRCC) is the most prevalent pathological type of renal cell carcinoma, contributing to around 80% of all kidney cancers^1,2^. Currently, surgical intervention is the sole primary therapeutic option for ccRCC^3^. Complete surgical excision, including either partial or radical nephrectomy, offers an opportunity for a cure for individuals with localised renal cell carcinoma^4^. However, up to 30% of those with localised ccRCC have reportedly experienced tumor recurrence after surgery and developed distant metastasis^5^. Moreover, approximately 25%–30% of patients with ccRCC presented with local progression and distant metastasis at the time of initial diagnosis^6^. The prognosis of metastatic ccRCC is dismal, with a 5-year survival rate of only 10%^7^. Therefore, identifying novel biomarkers and risk factors to establish accurate prognosis predictive models is critical for establishing successful therapeutic measures for ccRCC.

Cell death is a fundamental physiological process that regulates tissue homeostasis and development^8^. Therefore, the targeting of cell death-related pathways to eliminate tumor cells is a significant area of focus in cancer treatment^9^. Recently, Liu et al. reported a recently defined mode of regulated cell death termed ‘disulfidptosis’. It is worth noting that disulfidptosis differs from existing programmed cell death mechanisms, including apoptosis, necroptosis, ferroptosis, and pyroptosis. This distinct cell death mode is triggered by disulfide stress that results from the aberrant accumulation of intracellular disulfides under glucose starvation. Specifically, in cells expressing high levels of Solute carrier family 7 member 11 (SLC7A11) under glucose starvation, there is an increase in cystine uptake. The inadequate nicotinamide adenine dinucleotide phosphate (NADPH) supply further exacerbates the problem, leading to the depletion of NADPH and abnormal disulfide bonding in actin cytoskeleton proteins. Consequently, the actin network collapses, ultimately resulting in cell death^10^. This unique cell death mechanism is independent of ATP depletion or cystine crystal formation. It is resistant to conventional cell death inhibitors and remains unaffected by the downregulation of crucial ferroptosis/apoptosis genes^11^. Conversely, thiol oxidizers like diamide and diethyl maleate significantly enhance this mode of cell death^12^. Prior research has reported that SLC7A11 was overexpressed in renal cell carcinoma^13,14^, proposing that disulfidptosis performs a crucial function in ccRCC.

Non-coding RNA transcripts that are over 200 nucleotides in length are termed long non-coding RNAs (lncRNAs)^15^. Increasingly, research has determined an association between lncRNAs and ccRCC development and progression. Wang et al. noted that lncRNA MILIP enhanced ccRCC metastasis by linking YBX1 to the translational activation of Snai1^16^. Similarly, Liu et al. reported that lncRNA COL18A1-AS1 suppressed ccRCC advancement by inducing lipid browning through the miR-1286/KLF12 axis^17^. Moreover, multiple studies have provided evidence supporting the potential of lncRNAs as prognostic markers for predicting immunotherapy response and clinical outcomes^18,19^. Nonetheless, the prognosis-predictive value of disulfidptosis-associated lncRNAs in ccRCC is yet to be systematically explored. Therefore, the focus of this research is on assessing the involvement of disulfidptosis-related lncRNAs in ccRCC through bioinformatics.

## Materials and methods

### Process summary and data acquisition

**Figure 1:**
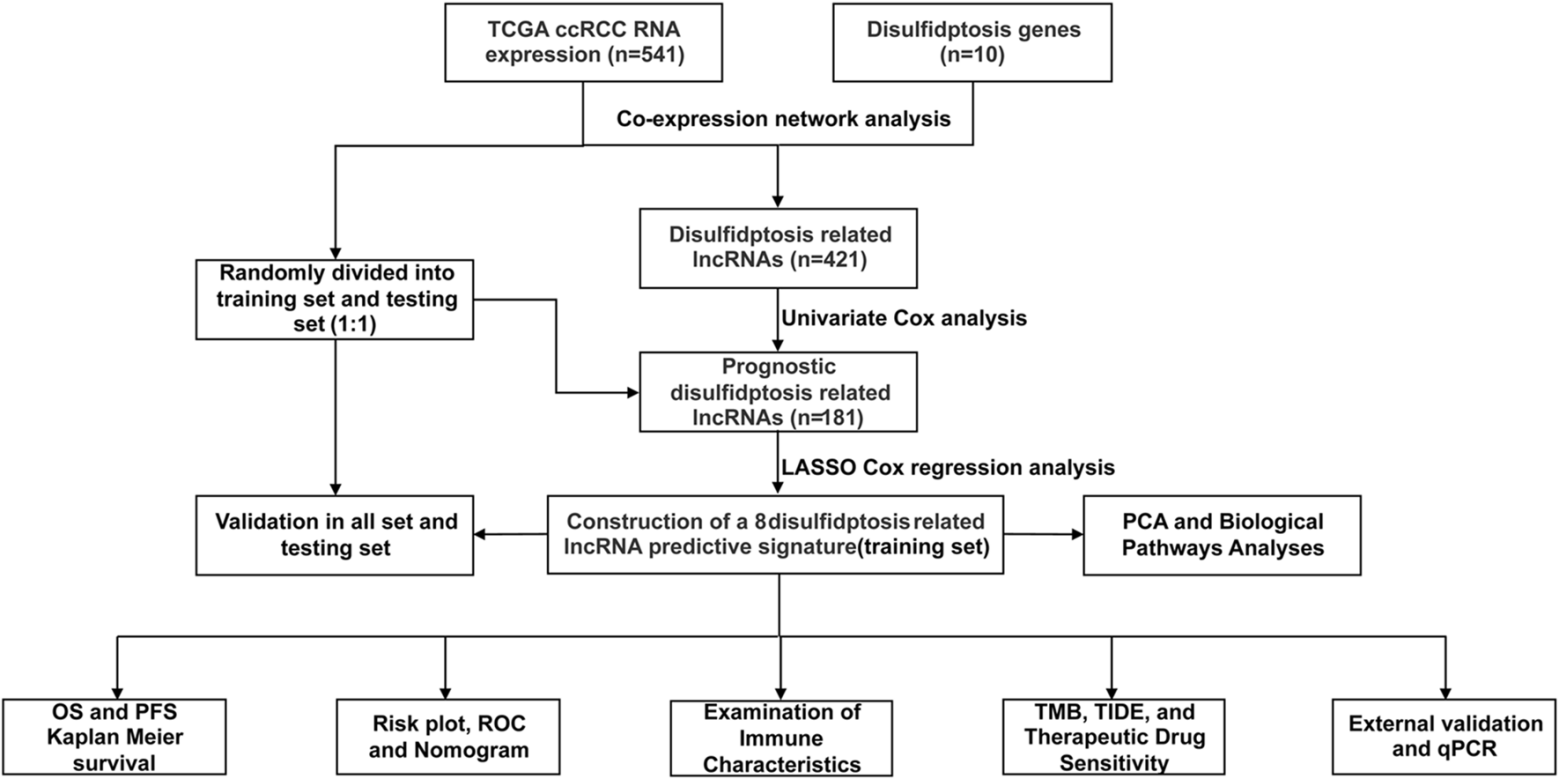
Flow chart of the present study.

The overall study workflow is presented in Fig. 1. A total of 10 disulfidptosis-related genes, namely OXSM, NDUFS1, GYS1, NDUFA11, LRPPRC, SLC3A2, NCKAP1, NUBPL, RPN1, and SLC7A11, was extracted from a previous study^10^. The transcriptome RNA sequencing data, somatic mutation, copy number variation data, and the clinical characteristics of individuals with ccRCC were accessed at the public database of TCGA on 3 December 2022 (https://portal.gdc.cancer.gov/repository). The study included 541 tumors and 72 normal tissue samples in total. Utilizing the Perl programming language (version: Strawberry-Perl-5.30.0; https://www.perl.org), the expression matrix and relevant clinical information were extracted.

### Identification of disulfidptosis-related lncRNAs

Based on the gene biotype file (GRCh39) downloaded from NCBI, lncRNAs were extracted. Pearson’s correlation analysis investigated the link between disulfidptosis-related genes and lncRNAs. Given the relevance of correlation coefficients in hierarchically characterizing the degree of correlation between parameters^20^, along with previous experience, lncRNAs with criteria of |Pearson R|> 0.4 and *p* < 0.001 were deemed as disulfidptosis-related lncRNAs^21^. The R packages ‘limma’, ‘dplyr’, ‘ggalluvial’, and ‘ggplot2’ were utilized to create a Sankey diagram illustrating the association of disulfidptosis genes with disulfidptosis-related lncRNAs^22^.

### Development and validation of the risk score model

TCGA was searched to retrieve the KIRC dataset, which was categorized randomly into training and internal testing sets at a ratio of 1:1 with the aid of the R ‘caret’. The fairness of the grouping was verified using a chi-square test. Table 1 exhibits the clinical features of both sets. The training set was employed for establishing the disulfidptosis-associated lncRNA signatures, while the testing set and the entire set were used to verify the signatures. Univariate Cox regression analysis of the training dataset was employed to determine the lncRNAs linked to prognosis (*p* < 0.05). Additionally, the R ‘glmnet’ was utilized to implement the Least Absolute Shrinkage and Selection Operator (LASSO) regression to determine the relevant lncRNAs linked to the prognosis of ccRCC individuals. The study utilized lambda.min as the threshold value for model selection. Ultimately, the lncRNAs filtered through further LASSO regression analysis were included in the multivariate Cox regression analysis to construct the optimal prognostic risk model using a stepwise regression method. The below-mentioned formula was utilized to calculate the risk score of every individual with ccRCC:

**Table 1:** Clinicopathological characteristics of ccRCC patients in training set and testing set.

Covariates	Type	Test	Train	*P-*value
Age	< = 65	173 (65.04%)	176 (66.17%)	0.8552
	> 65	93 (34.96%)	90 (33.83%)	
Gender	Female	95 (35.71%)	92 (34.59%)	0.8559
	Male	171 (64.29%)	174 (65.41%)	
Grade	G1	6 (2.26%)	8 (3.01%)	0.4461
	G2	114 (42.86%)	114 (42.86%)	
	G3	98 (36.84%)	108 (40.6%)	
	G4	44 (16.54%)	32 (12.03%)	
	Unknow	4 (1.5%)	4 (1.5%)	
Stage	Stage I	129 (48.5%)	137 (51.5%)	0.9014
	Stage II	30 (11.28%)	27 (10.15%)	
	Stage III	63 (23.68%)	60 (22.56%)	
	Stage IV	43 (16.17%)	40 (15.04%)	
	Unknow	1 (0.38%)	2 (0.75%)	
T	T1	133 (50%)	139 (52.26%)	0.7337
	T2	36 (13.53%)	33 (12.41%)	
	T3	93 (34.96%)	87 (32.71%)	
	T4	4 (1.5%)	7 (2.63%)	
M	M0	210 (78.95%)	211 (79.32%)	0.6796
	M1	42 (15.79%)	37 (13.91%)	
	Unknow	14 (5.26%)	18 (6.77%)	
N	N0	125 (46.99%)	115 (43.23%)	0.0658
	N1	4 (1.5%)	12 (4.51%)	
	Unknow	137 (51.5%)	139 (52.26%)	

$$\mathrm{Risk score}\hspace{0.17em}=\hspace{0.17em}\sum_{i=1}^{n}Expr\left(lncRNAi\right)* Coef(lncRNAi).$$

*Coef(lncRNAi)* represents the regression coefficient of the corresponding lncRNA. *Expr (lncRNAi)* represents the normalized expression level for each lncRNA, and the unit of the risk score is FPKM^23^. The samples were categorized as per their median of the training set into low-risk and high-risk groups. Kaplan–Meier (KM) curves compared the differences in overall survival (OS) and progression-free survival (PFS) between the two groups. The diagnostic value of the risk model was validated through a Risk Plot analysis utilizing the R ‘pheatmap’. Furthermore, multivariate and univariate Cox regression analyses were conducted to investigate whether the constructed risk model could act as an independent risk factor, considering various clinical signatures (age, gender, grade, and stage), for individuals with ccRCC. Utilizing the R ‘survival’, ‘survminer’, ‘rms’, ‘caret’, ‘glmnet’, and ‘timeROC’, receiver operating characteristic (ROC) curves were generated to calculate the area under the curve (AUC) and a consistency index (C-index) was employed to evaluate the predictive ability of the model. The predictive nomogram was established for the prediction of 1-, 3- and 5-year survival rates for individuals with ccRCC, and the calibration curve was employed to examine the difference between predicted and actual observed values.

### Principal component analysis (PCA) and functional enrichment analysis

PCA is the most frequently used statistical tool for dimensionality reduction and data analysis^24^. Herein, PCA evaluated the grouping ability of all genes, disulfidptosis genes, disulfidptosis-related lncRNAs, and the identified risk lncRNAs.Concerning the genes depicting differential expression between the two risk groups (low- and high-risk), the R ‘clusterProfiler’ was utilized for Gene Ontology (GO) and Kyoto Encyclopaedia of Genes and Genomes (KEGG) analyses^25–27^. FDR < 0.05 and *p* < 0.05 values were utilized to define the significantly enriched biological processes and pathways. Additionally, gene set enrichment analysis (GSEA) was executed to determine the significantly different functional phenotypes between the risk groups. Furthermore, gene sets linked to different hallmarks were accessed at the Molecular Signatures Database (MSigDB, http://software.broadinstitute.org/gsea/msigdb/). Its subset C7: immunologic signature gene set was also simultaneously employed. Single sample gene set enrichment analysis (ssGSEA) was implemented to profile the overall immune and stromal infiltration levels in patients with ccRCC. Utilizing the R package ‘GSVA’, the gene set variation analysis (GSVA) was conducted to derive the enrichment score for each gene signature, with *p* < 0.05 indicating statistical significance.

### Immune cell infiltration and immune score analysis

The association of the established model with the immune infiltration status was examined through the CIBERSORT algorithm. The process involved assessing the percentage of immune cell subsets in each TCGA-KIRC sample^28^. Additionally, the matrix and immune scores were computed using the ESTIMATE method to evaluate tumor purity and cell type distribution in the tumor microenvironment (TME)^29^.

### Tumor mutation burden (TMB) and tumor immune dysfunction and exclusion (TIDE)

Using Pearl, the data of somatic mutations were acquired from the TCGA. Afterward, the R ‘maftools’ package was utilized for evaluation and integration of the TCGA data. The variations in TMB and survival rates between the two risk groups were compared. The response to immune checkpoint blockade (ICB) therapy was examined through the online tool TIDE (http://tide.dfci.harvard.edu/login/)^30^. Furthermore, the TIDE scores between the subgroups were compared using the ‘ggpubr’ R package.

### Drug sensitivity estimation

The R package ‘oncoPredict’ was utilized for predicting the IC50 values of drugs that were available as a therapeutic measure for ccRCC in the two risk groups^31^. The anti-cancer drug sensitivity databases used were Genomics of Drug Sensitivity in Cancer (GDSC) and Cancer Therapeutics Response Portal (CTRP), both of which were included in R ‘oncoPredict’.

### Cell culture and qPCR

Normal human renal cell line HK-2 cells were acquired from the American Type Culture Collection (Manassas, USA) and cultured in DMEM/F12 medium (Gibco, Brazil) with 10% foetal bovine serum (FBS, Biological Industries, Israel). The human ccRCC cell lines 786-O and ACHN were acquired from the National Collection of Authenticated Cell Cultures, Chinese Academy of Sciences (Shanghai, China). The iCell Bioscience lnc (Shanghai, China) supplied the human ccRCC cell line 769-P. In addition, 10% and 1% penicillin–streptomycin (Solarbio, China)-supplemented RPMI-1640 medium (Invitrogen, USA) was utilized for the growth of 786-O and 769-P cells. ACHN cells were maintained in MEM (iCell Bioscience lnc, China) with 10% FBS and 1% penicillin–streptomycin. The cells were incubated in an incubator at 37 °C under humid conditions and 5% CO_2_.

Extraction of the total RNA from HK-2 and ccRCC cells was executed through the RNAeasy™ animal isolation kit with spin columns (Beyotime, China). The reverse transcription kit (FSQ-101, TOYOBO, Japan) was utilized to synthesize the complementary DNA (cDNA) in a 10 μL qPCR reaction system (A301, Genstar, China). The system comprised cDNA templates (3 μL), forward and reverse primers (0.5 μL), 2 × RealStar Green Fast Mixture (5 μL), ROX Reference Dye (0.2 μL), and RNase-free H2O (0.8 μL). The reactions were repeated thrice employing a 7500 Real-Time PCR System (Thermo Fisher Scientific, USA). The amplification procedure involved three steps; initiation at 95 °C (2 min), along with subsequent 95 °C (15 s), 60 °C (30 s), and 72 °C (30 s) for 40 cycles. Employing the 2^−△△CT^ method, the relative expression levels of lncRNAs were normalized to the housekeeping gene RPL13A. Table 2 depicts the primer sequences. Per each cDNA sample, three assays were executed.

**Table 2:** Sequence of primers for qPCR.

Primer name	Direction	Primer sequence 5’—3’
LINC00886	Forward	TCCAGGCTTTCTTGCACACA
LINC00886	Reverse	GTGGGCTTGACAGGAAAGGT
SPINT1-AS1	Forward	GGATCCAATGTCTTTAGCCAAC
SPINT1-AS1	Reverse	ACTGCAGTCAAGGTGTCAG
RPL13A	Forward	CCTGGAGGAGAAGAGGAAAGAGA
RPL13A	Reverse	TTGAGGACCTCTGTGTATTTGTCAA

### Statistical analysis

R 4.2.1 was utilized for conducting all statistical analyses. Additionally, pairwise comparison was conducted by means of the Wilcoxon test. Furthermore, KM analysis and log-rank test comparatively assessed OS between the samples. Statistical significance was deemed to be attained at two-sided *p* < 0.05.

## Results

### Identification of disulfidptosis-related lncRNAs

**Figure 2:**
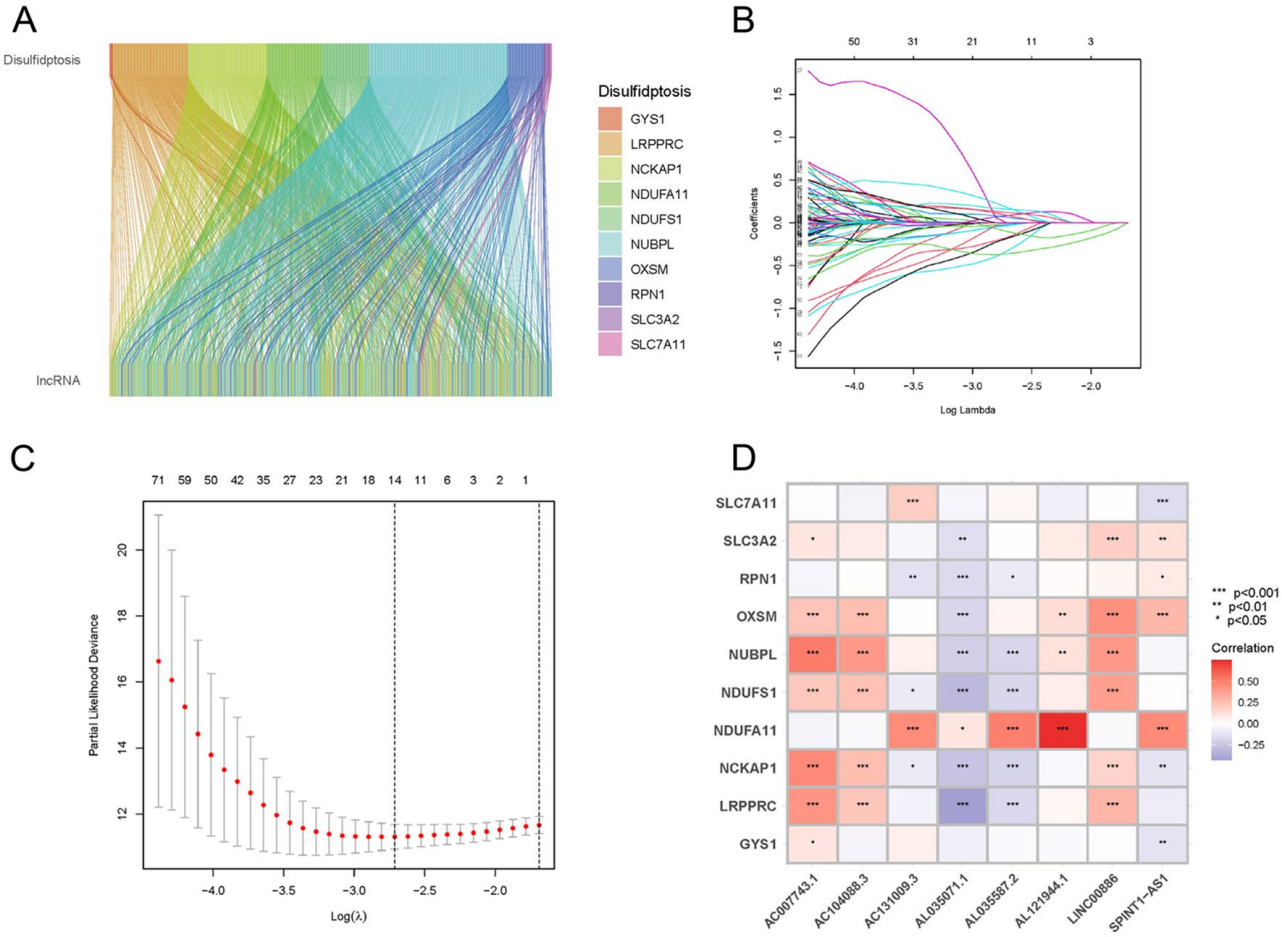
Identification of disulfidptosis-related lncRNAs and prognostic value preparation in ccRCC. (**A**) Sankey diagram for disulfidptosis genes and disulfidptosis-related lncRNAs. (**B**) LASSO coefficient profiles of 181 disulfidptosis-related lncRNAs. (**C**) Selection of tuning parameter lambda in the LASSO Cox regression model using ten-fold cross-validation. (**D**) Heatmap for the correlation between disulfidptosis genes and 8 disulfidptosis-related lncRNAs.

Overall, 431 lncRNAs were screened in the ccRCC samples by means of Pearson correlation analysis (|Pearson R|> 0.4 and *p* < 0.001). A Sankey map (Fig. 2A) and Table S1 illustrated the corresponding relationship between the 10 disulfidptosis genes and 431 disulfidptosis-related lncRNAs.

### Establishment of prognostic disulfidptosis-related lncRNA signature

Univariate Cox regression analysis was conducted, yielding 181 differentially expressed prognostic disulfidptosis-related lncRNAs (Table S2). The training set underwent LASSO analysis to determine the lncRNAs with the highest prognosis-predictive values (Fig. 2B,C). Ultimately, 14 lncRNAs were filtered with eight being imported into the multi-Cox proportional risk model. The model formula employed is as follows: risk score = SPINT1-AS1 × (− 0.398170) + AL121944.1 × (− 1.366648) + AC131009.3 × (0.312254) + AC104088.3 × (− 0.932196) + AL035071.1 × (0.507753) + LINC00886 × (− 0.279419) + AL035587.2 × (0.891684) + AC007743.1 × (− 0.351352). To explore the expression correlation between the eight lncRNAs and 10 disulfidptosis genes, a correlation heatmap was generated based on TCGA samples (Fig. 2D).

### Validation of prognostic disulfidptosis-related lncRNA signature

**Figure 3:**
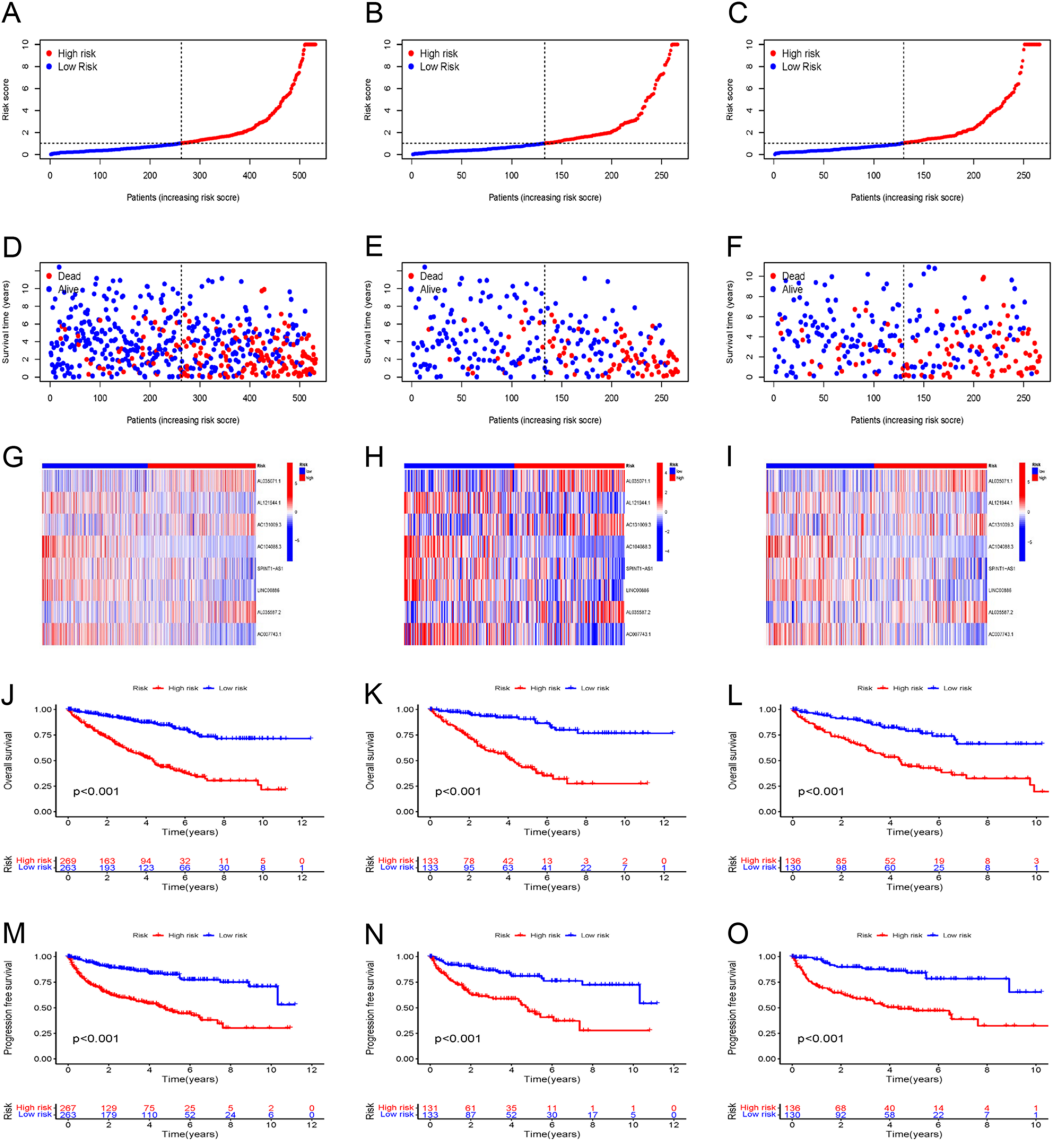
Prognostic value of the risk model in the train, test, and entire set. (**A**–**C**) Risk score distribution of patients in the low-risk and high-risk groups. (**D**–**F**) Survival status and time in the low-risk and high-risk groups. (**G**–**I**) Hierarchical clustering analysis of 8 disulfidptosis-related lncRNAs between the low-risk and high-risk groups. (**J**–**L**) Kaplan–Meier survival curves of OS between low-risk and high-risk groups. (**M**–**O**) Kaplan–Meier survival curves of progression-free survival between low-risk and high-risk groups.

**Figure 4:**
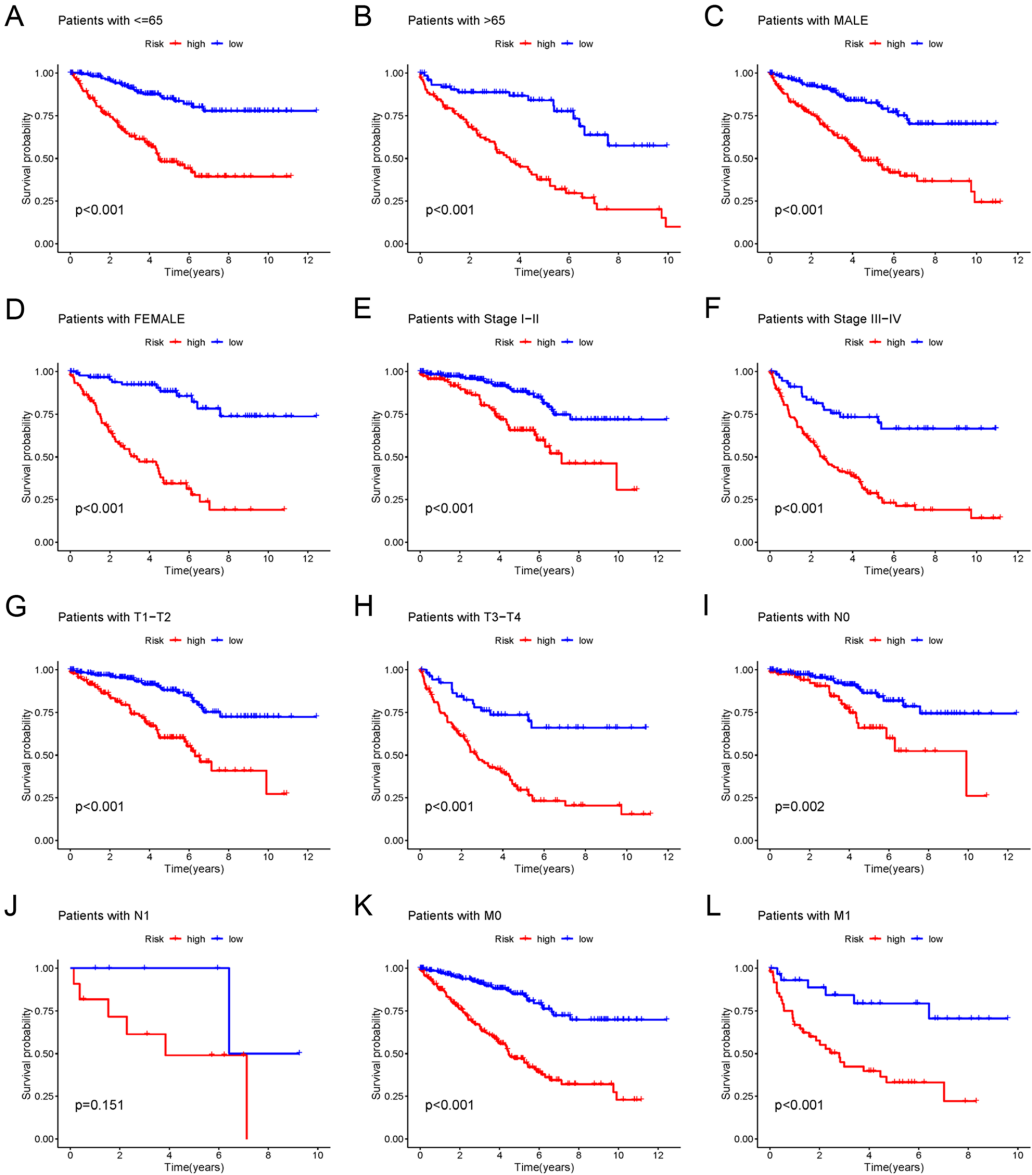
Kaplan–Meier survival curves for low-risk and high-risk populations by different clinical variables. (**A**,**B**) Survival curve after grouping according to age (≤ 65, > 65). (**C**,**D**) Survival curve after grouping according to sex (male, female). (**E**,**F**) Survival curve after grouping according to clinical stages (stage1-2, stage3-4). (**G**,**H**) Survival curve after grouping according to T stage (T1-2, T3-4). (**I**,**J**) Survival curve after grouping according to N stage (N0, N1). (**K**,**L**) Survival curve after grouping according to M stage (M0, M1).

The signature was evaluated for its predictive capability through a risk plot and KM survival analyses. The R ‘heatmap’ was utilized to map the risk plot. The survival outcome, relative expression, and risk score distributions of the eight disulfidptosis-related lncRNAs were assessed for both risk groups (low- and high-risk) (Fig. 3A–I). The analysis revealed a remarkably shorter OS for the individuals at increased risk in the entire set, training, and testing sets in contrast with the low-risk group individuals (All *p* < 0.001) (Fig. 3J–L). Moreover, the high-risk group depicted remarkably reduced PFS in contrast with the low-risk group (All *p* < 0.001) (Fig. 3M–O). Moreover, Kaplan–Meier plots were generated after grouping samples as per sex, age, stage, T-, N- and M-stages (Fig. 4A–L). Except for the N1 stage, OS rates in the high-risk subgroups were greater than that in the low-risk subgroups. Although a similar trend was observed for the N1 stage, the results lacked any statistical significance (*p* = 0.151). This could be attributed to the small sample size. The resulting data are indicative of the potential that the disulfidptosis-related lncRNA signature holds concerning its predictive ability regarding the prognosis of individuals with ccRCC with various clinicopathological factors.

**Figure 5:**
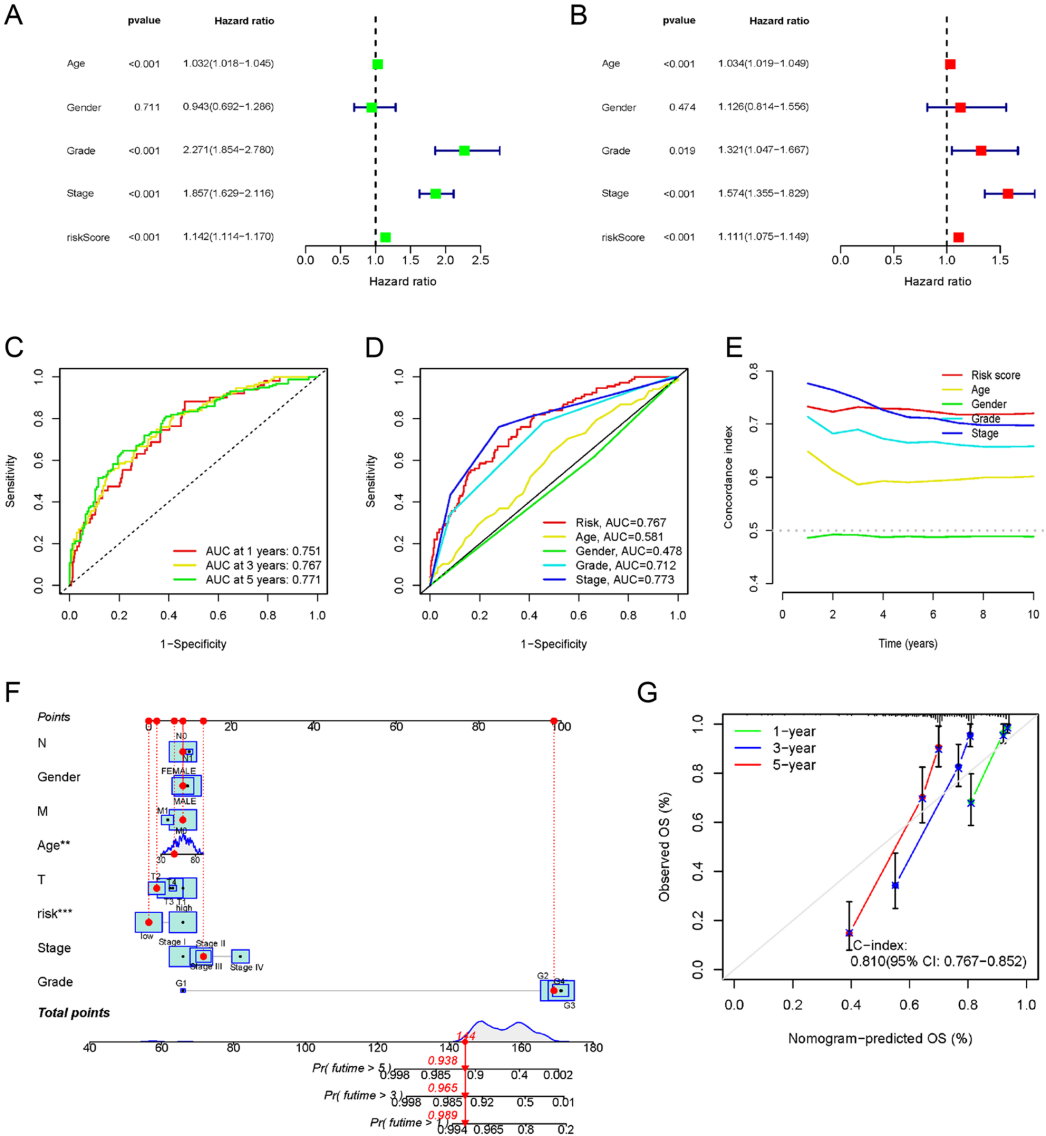
Independent prognostic analysis and further validation of the risk model. Forest plots of (**A**) univariate and (**B**) multivariate Cox regression analysis showed the effects between clinical characteristics (including the risk signature) and OS. (**C**) Time-dependent ROC curves of OS at 1-, 3- and 5-year. (**D**) Predictive accuracy of the risk model compared with clinicopathologic characteristics. (**E**) Concordance index of the risk model and other clinical information. (**F**) Nomogram combining the risk signature and clinical factors. (**G**) Calibration curves for the nomogram-predicted OS at 1, 3, and 5 years (C-index = 0.810, *p* < 0.001).

Afterward, univariate and multivariate Cox regression analyses were conducted to assess whether the prognostic characteristics of the signature remained independent of clinical stage, sex, age, and TNM stage (Fig. 5A,B). The resulting data exhibited that the established signature remained an independent prognosis-predictive factor. Moreover, ROC analysis validated the risk model concerning its predictive efficiency. The AUC was 0.751, 0.767, and 0.771 for the 1-, 3- and 5-year ROCs, respectively (Fig. 5C). It is worth noting that in terms of predicting the patient long-term survival, the risk score outperformed most clinical factors as depicted through comparative ROC curves (Fig. 5D). Moreover, the concordance index of the risk signature and other clinical data suggested that the signature could serve as a reliable reference index in clinical settings (Fig. 5E). Furthermore, to provide clinically convenient prognostic predictions for individual patients, a risk score-based nomogram was developed (Fig. 5F). The predicted outcomes were shown to have a high level of agreement with the nomogram through calibration curves (Fig. 5G).

### PCA and biological pathways analyses

**Figure 6:**
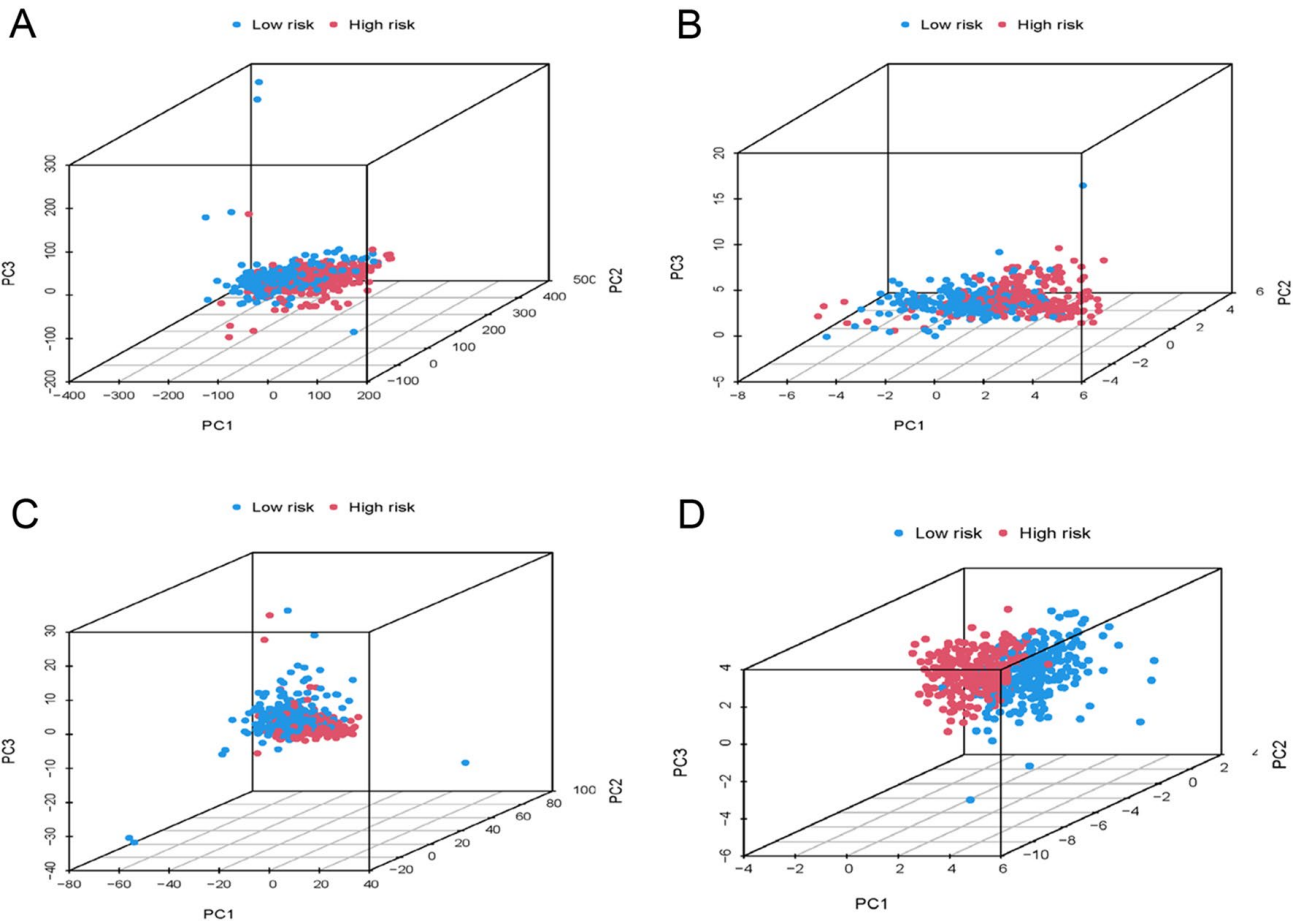
PCA between low-risk and high-risk groups. (**A**) PCA of all genes. (**B**) PCA of disulfidptosis genes. (**C**) PCA of disulfidptosis-related lncRNAs. (**D**) PCA of 8 risk lncRNAs.

PCA validated the capacity of the signature to distinguish between low- and high-risk groups as per the identified eight disulfidptosis-related lncRNAs. The low- and high-risk sample distribution based on all genes, disulfidptosis genes, disulfidptosis-related lncRNAs, and the identified risk lncRNAs is presented in Fig. 6A–D. The outcomes implied that the risk model was capable of accurately distinguishing between patients at high or low risk.

**Figure 7:**
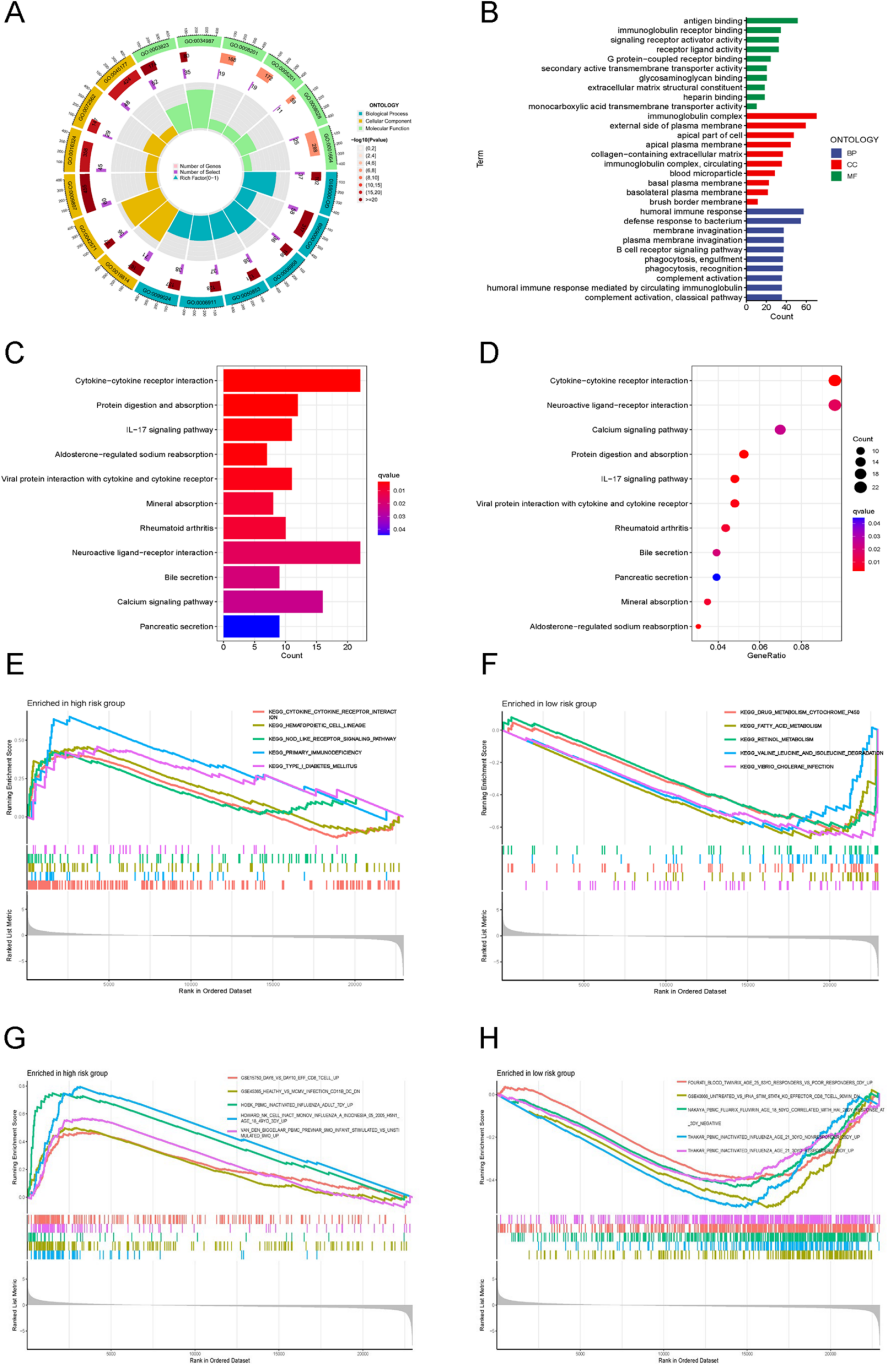
Functional analysis of the risk model. (**A**,**B**) GO analysis demonstrated the richness of molecular biological processes (BP), cellular components (CC), and molecular functions (MF). (**C**,**D**) KEGG pathway analysis showed the significantly enriched pathways. (**E**,**F**) GSEA analysis based on KEGG pathway database of high-risk group and low-risk group. (**G**,**H**) GSEA analysis based on C7 gene set of high-risk group and low-risk group.

Various analyses were conducted for a detailed assessment of the biological functions linked to the eight disulfidptosis-related lncRNA signatures. GO enrichment analysis revealed the enrichment of the differentially expressed genes primarily in the development of immune responses, such as antigen binding and immunoglobulin complex (Fig. 7A,B). KEGG analysis exhibited the enrichment of differential genes primarily in certain signaling pathways such as IL-17 and cytokine − cytokine receptor interaction (Fig. 7C,D). Additionally, GSEA indicated that the five leading pathways enriched in the two risk groups were distinct from each other (Fig. 7E–H).

### Evaluation of immune characteristics based on the risk model

**Figure 8:**
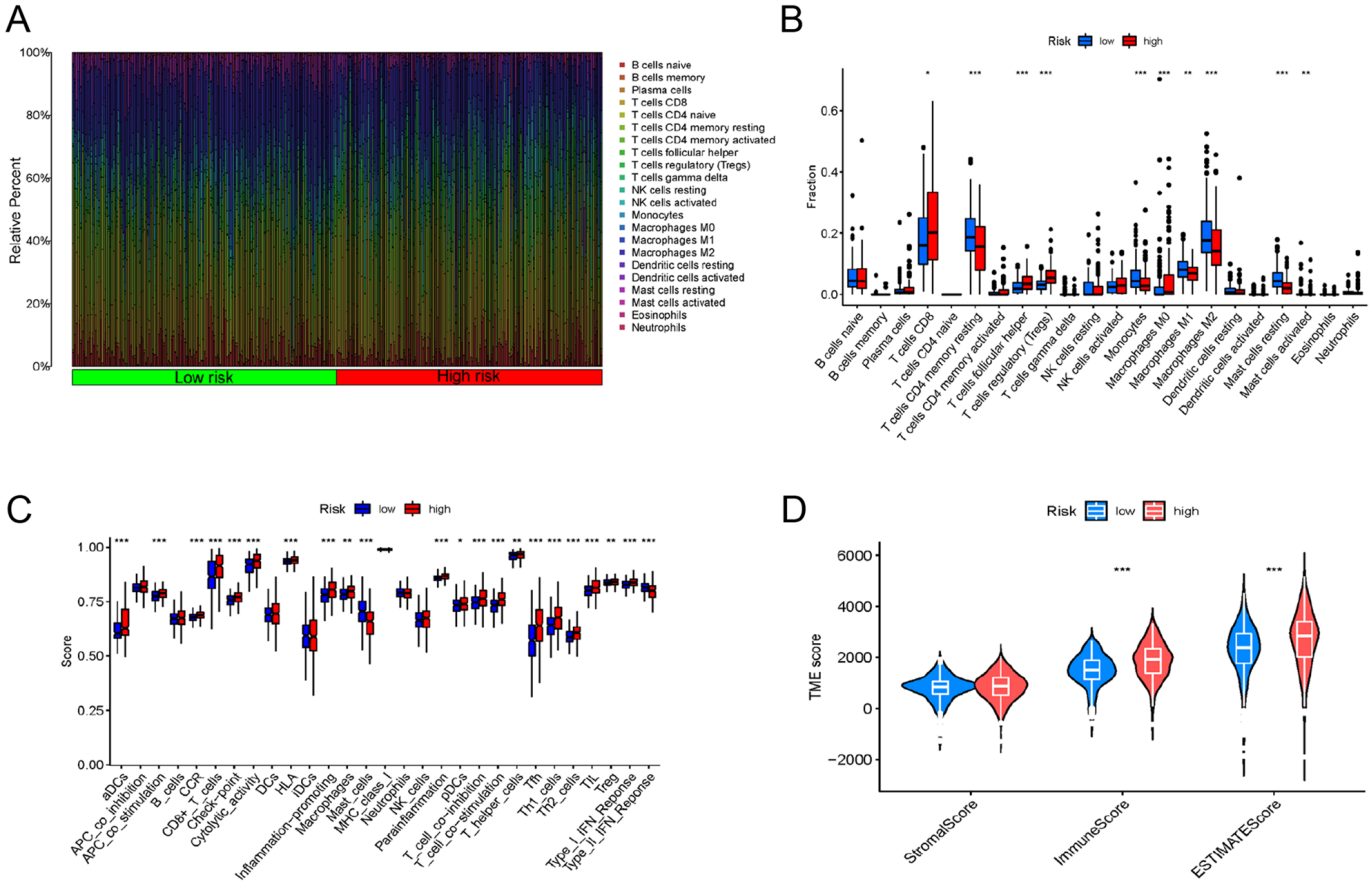
Differences in the tumor immune microenvironment between the low- and high-risk groups. (**A**) The abundance ratios of immune cells in the ccRCC samples. (**B**) Differentially expressed immune cells in the high- and low-risk score groups. (**C**) Immune function analysis in the high-risk and low-risk score groups. (**D**) Violin diagram comparing StromalScore, ImmuneScore and ESTIMATEScore between the low-risk and high-risk groups.

R ‘CIBERSORT’ was utilized to calculate the infiltration of immune cells in the TCGA cohort. Figure 8A displays the relative abundance of the 22 types of immune cells in patients with ccRCC. The immune cells depicting differential expression between the two risk score groups, such as follicular helper T cells, CD8 T cells, Tregs, resting CD4 memory T cells, monocytes, M2 macrophages, M1 macrophages, M0 macrophages, mast cells (resting and activated), were identified via CIBERSORT R (Fig. 8B). Subsequent exploration of immune function revealed that the two groups varied remarkably in terms of several immune functions. These included aDCs, cytolytic activity, CCR, CD8^+^ T cells, APC co-stimulation, mast cells, checkpoint, inflammation promotion, HLA, para-inflammation, macrophages, pDCs, IFN response (type I and II), T cell co-inhibition and co-stimulation, helper T cells, Tfh, Th2 cells, Th1 cells, TIL, and Treg (Fig. 8C) (*p* < 0.05). Among these immune functions, only type II IFN response and mast cells were remarkably suppressed in the high-risk group. With regard to the TME scores, the individuals at high risk depicted increased values for immune and ESTIMATE scores than the individuals at low risk. However, no notable variations in stromal scores were observed (Fig. 8D).

### TMB and TIDE

**Figure 9:**
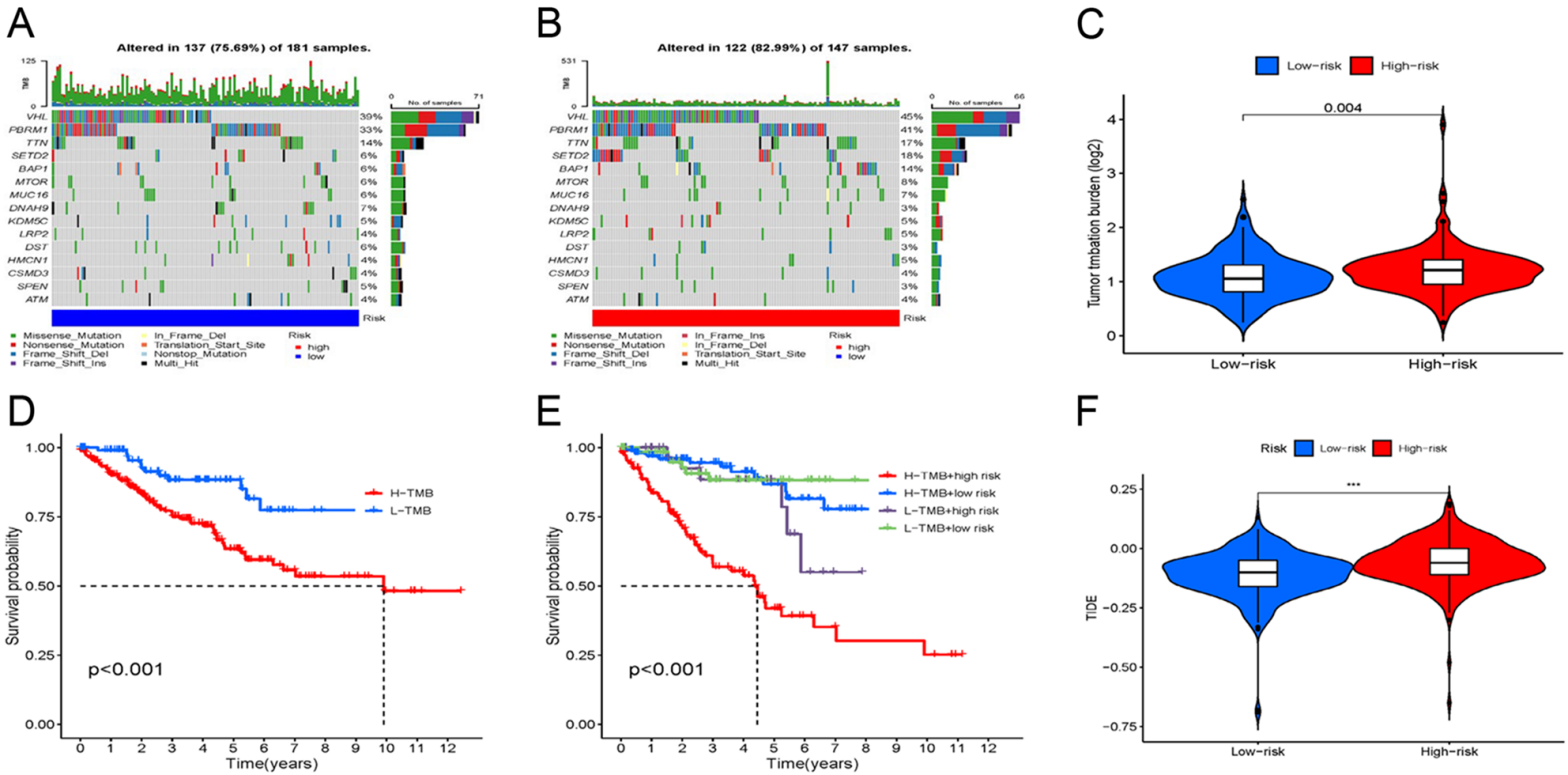
Relationship of model scores to TMB and TIDE. (**A**,**B**) Waterfall plots of somatic mutation characteristics in the two groups. (**C**) TMB between the low-risk and high-risk groups. (**D**) Kaplan–Meier analysis of the effect of TMB status on OS. (**E**) Kaplan–Meier analysis for OS of patients categorized by combing TMB status and risk score. (**F**) TIDE scores between the two groups.

The R ‘maftools’ was employed to assess both groups for the frequency of mutations and TMB. For visualization purposes, the 15 leading genes concerning mutation frequency were selected. The waterfall plot (Fig. 9A,B) filtered the five leading mutated genes as *VHL, PBRM1, TTN, SETD2,* and *BAP1* in the ccRCC samples. Among them, *VHL, PBRM1, SETD2*, and *BAP1* were the most frequently mutated genes in ccRCC. Furthermore, in addition to counting the number of variants in each sample, the ccRCC mutation types were visually represented using different colors in box plots. Most genes in the group with high risk depicted a higher frequency of mutations than the group with low risk. Furthermore, TMB was assessed in both groups. The violin plot revealed that the group with high risk was remarkably linked to increased TMB (*p* = 0.004) (Fig. 9C). Additionally, a KM plot was generated between high- and low-TMB groups. The individuals in the latter group depicted a more favourable prognosis in contrast with those in the former group (Fig. 9D). Furthermore, on combining TMB with the risk score of a patient, a worse prognosis was linked to the high-risk and TMB-high group (*p* < 0.001) (Fig. 9E). Finally, the effect of immunotherapy in high-risk and low-risk patients was assessed. The former group had a greater TIDE score relative to the latter one, implying that the potential for immune escape was greater in the individuals at high risk, leading to less effective immunotherapy (*p* < 0.001) (Fig. 9F).

### Screening potential anticancer drugs for ccRCC

**Figure 10:**
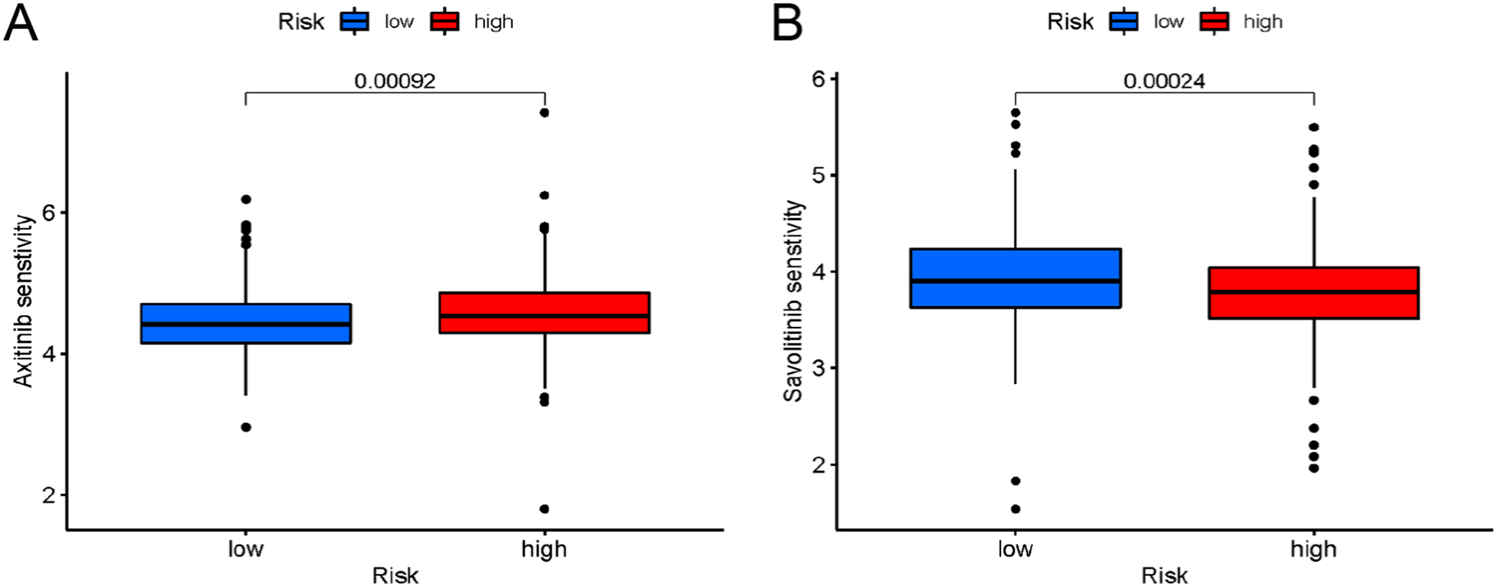
Drug sensitivity. (**A**) Axitinib was more sensitive in the low-risk group. (**B**) Savolitinib was more effective in the high-risk group.

The ‘oncoPredict’ R package was utilized to screen 78 anti-cancer drugs whose sensitivity significantly correlated with the model. Of these drugs, increased sensitivity was depicted by 51 drugs in the high-risk group (Fig. S1) and 27 drugs in the low-risk group (Fig. S2). Specifically, axitinib was observed to depict increased sensitivity in the group with low risk (Fig. 10A), while savolitinib exhibited higher sensitivity in the group with low risk (Fig. 10B).

### External validation of disulfidptosis-related lncRNAs

**Figure 11:**
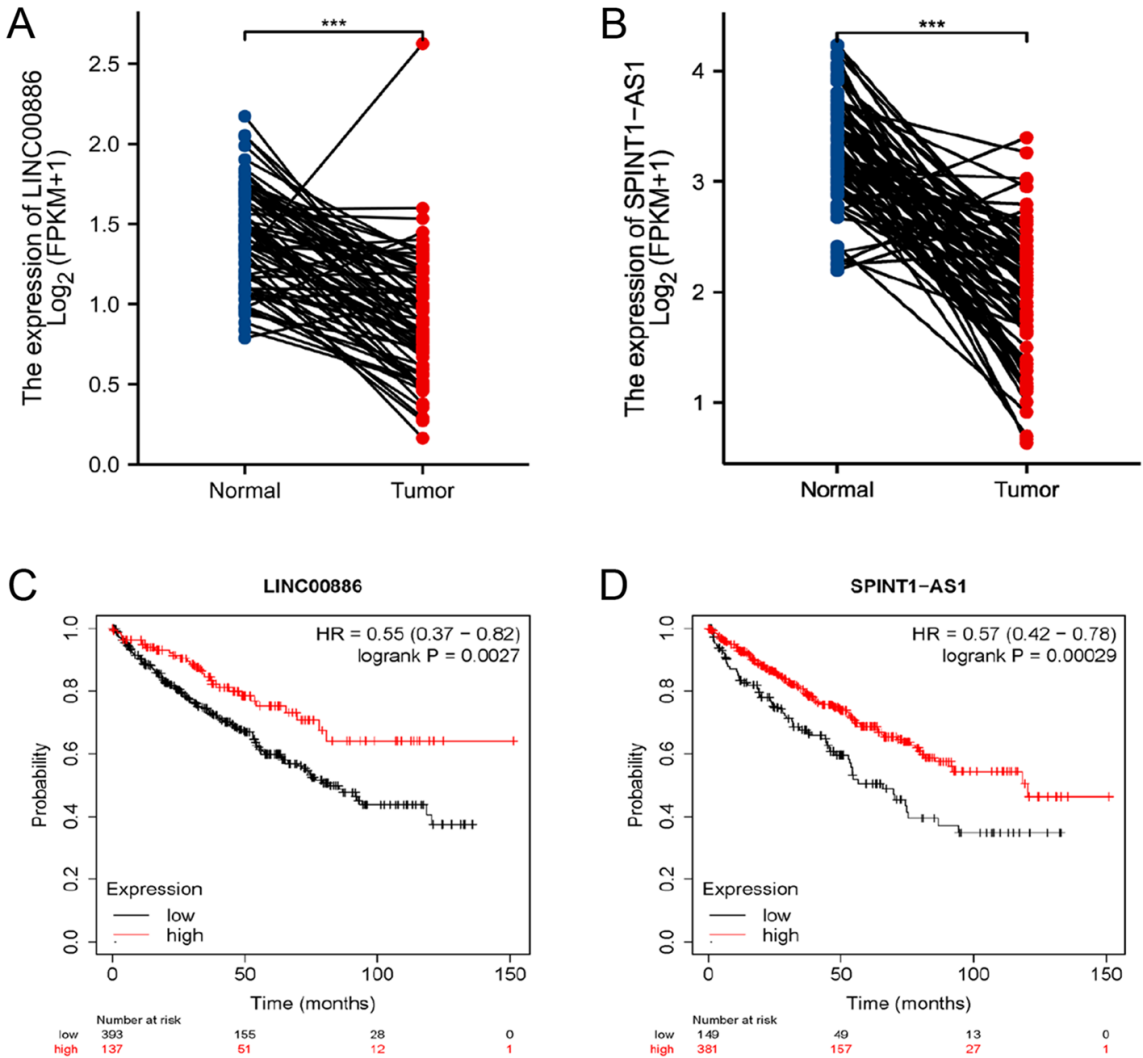
External validation of disulfidptosis-associated lncRNAs. (**A**,**B**) The expression of LINC00886 and SPINT1-AS1 in tumor tissues and paired normal tissues of TGCA database. (**C**,**D**) OS analysis of LINC00886 and SPINT1-AS1 in the Kaplan–Meier Plotter datasets.

To validate this risk model, the differential expression of LINC00886 and SPINT1-AS1 in both tumor and paired normal tissues was examined utilizing information from TCGA. The expression of both genes was downregulated in tumor tissues (Fig. 11A,B). Afterward, the prognostic significance of LINC00886 and SPINT1-AS1 was assessed through the external KM plotter (https://kmplot.com/analysis/). The analysis revealed that LINC00886, acting as a favourable prognostic indicator, was remarkably linked to OS (hazard ratio (HR) = 0.55 (0.37–0.82), Log-rank *p* = 0.0027) (Fig. 11C). Similarly, SPINT1-AS1, also a marker of good prognosis, was remarkably linked to OS (HR = 0.57 (0.42–0.78), Log-rank *p* = 0.00029) (Fig. 11D). The resulting data of the validation of external datasets were congruent with the outcomes of this research.

### Validation of disulfidptosis-related lncRNAs using qPCR

**Figure 12:**
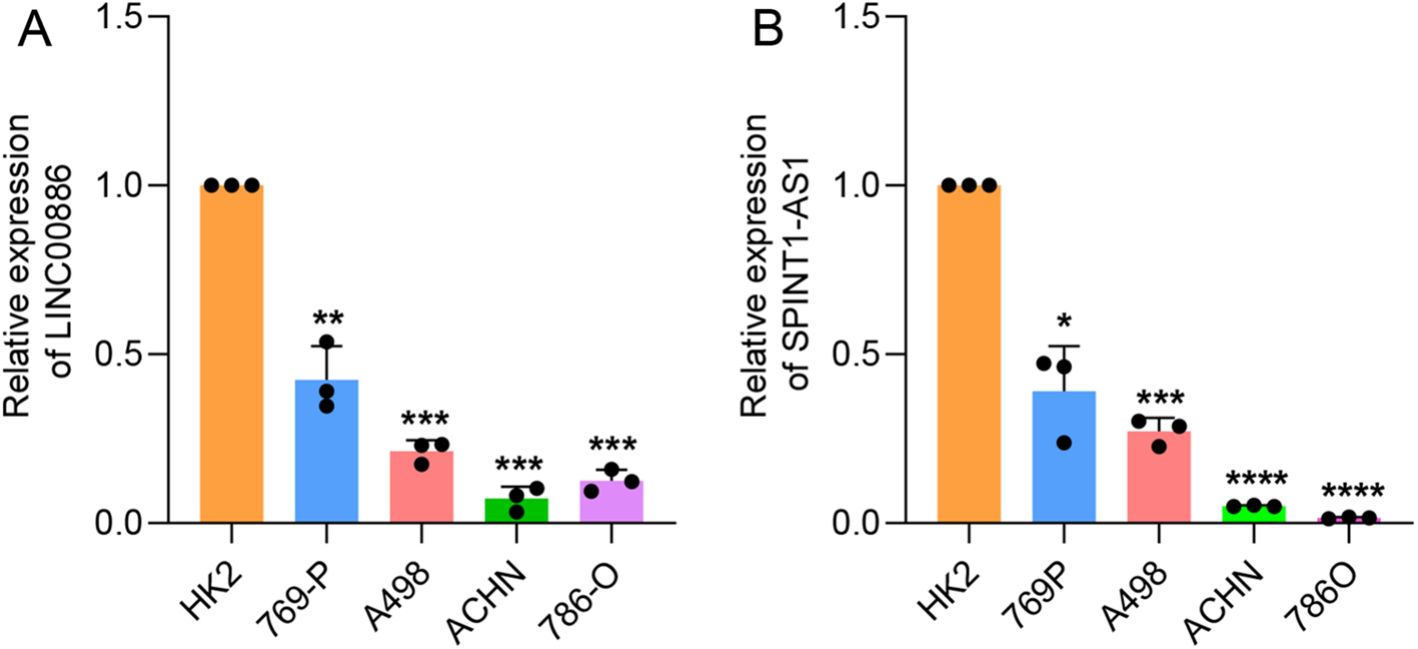
Expression levels of hub risk disulfidptosis-associated lncRNAs in ccRCC cell lines and HK-2 cell. (**A**) The expression levels of LINC00886 measured by qPCR. (**B**) The expression levels of SPINT1-AS1 measured by qPCR. The significant differences from the HK-2 were indicated with star (*p < 0.05; **p < 0.01; ***p < 0.001, ****p < 0.0001).

The predictive values of the disulfidptosis-related lncRNA model were validated by verifying the hub disulfidptosis-associated lncRNA expression in the aforementioned cell lines using qPCR. The reduced expression levels of LINC00886 and SPINT1-AS1 were observed in ccRCC cells in contrast with normal renal cell line HK-2 cells (Fig. 12A,B), which was consistent with previously reported results.

## Discussion

The regulated mode of cell death, disulfidptosis, is a recently defined mode that results from the aberrant accumulation of intracellular cystine and other disulfides^10^. Extracellular cystine is primarily transported into the cell by the cell membrane surface molecule SLC7A11/xCT (functional subunit of the cysteine/glutamate reverse transporter System xc-)^32,33^. Subsequently, it is quickly reduced to cysteine for glutathione (GSH) synthesis, a process that relies heavily on NADPH generation through the glucose-pentose phosphate pathway^34^. GSH plays a crucial role in activating or inducing antioxidant enzymes, maintaining normal protein function, and neutralizing cytotoxic substances^35^. Moreover, SLC7A11 is often overexpressed in tumors, and cancer cells depend on SLC7A11-mediated cystine uptake to regulate redox homeostasis and promote cell survival. However, excessive cystine accumulation can be cytotoxic^13^. Thus, for homeostasis, cancer cells are compelled to use NADPH to rapidly convert cystine into cysteine. NADPH is mainly supplied by glucose; therefore, cutting off the glucose supply induces cystine accumulation and leads to cell death. Gan et al. explored the mechanism behind this phenomenon and finally proposed an undescribed form of cell death called disulfidptosis. Their study disclosed that in malignant cells with elevated expression levels of SLC7A11, under conditions of glucose depletion, there was a significant accumulation of disulfide molecules. This led to abnormalities in the disulfide bonding between actin cytoskeletal proteins. As a consequence, the organization of these proteins was disrupted, leading to the collapse of the actin network and ultimately cell death^10^. The discovery of disulfidptosis death opens up new possibilities for cancer treatment, and further understanding of the underlying mechanism of this process could provide additional targets for cancer therapy.

Previous studies have reported on the crucial role that lncRNAs play in regulated cell death. Cheng et al. reported that lncRNA SNHG16 inhibits the apoptotic process in ccRCC cells through its interactions with miR-1301-3p to enhance STARD9 expression^36^. Similarly, Lv et al. elaborated that lncRNA TUG1 promotes the proliferation of cells and suppresses the apoptotic processes and autophagy in ccRCC via the miR-31-5p/FLOT1 axis^37^. Furthermore, Lai et al. developed a prognostic signature using ferroptosis-associated lncRNAs for the prediction of prognosis and immune response in ccRCC^38^. These findings suggest that exploring the association between lncRNAs and cell death could provide insights into the potential processes linked to the progression of tumors. Nevertheless, the role of lncRNAs in disulfidptosis remains unexplored in ccRCC.

A signature for predicting the prognostic status of individuals with ccRCC was developed utilizing the disulfidptosis-associated lncRNAs. In this study, 181 disulfidptosis-related lncRNAs associated with prognosis were obtained utilizing the univariate Cox regression analysis. Using LASSO and multivariate Cox regression analysis, eight disulfidptosis-related lncRNAs that were remarkably linked to OS were identified (SPINT1-AS1, AL121944.1, AC131009.3, AC104088.3, AL035071.1. LINC00886, AL035587.2, and AC007743.1). Using these eight lncRNAs, a disulfidptosis-related lncRNA signature was developed for prognosis prediction of individuals with ccRCC. Moreover, one of these lncRNAs, SPINT1-AS1, has been associated with multi-cancer progression, including breast, cervical, and colorectal cancers and oesophageal squamous cell carcinoma^39–42^. Additionally, SPINT1-AS1 was also used to construct a nine-redox-related lncRNA signature for ccRCC^43^. Overall, SPINT1-AS1 depicts promise as a therapeutic target for cancer therapy. LINC00886 is considered a tumor suppressor factor with upregulated expression across diverse cancers, including laryngeal squamous cell carcinoma and oesophageal squamous cell carcinoma^44,45^. AC131009.3 was also used to develop an individualised clinical prognostic index based on ubiquitination-related lncRNA in patients with ccRCC^46^. Similarly, AC007743.1 was involved in the development of a novel pyroptosis-related lncRNA signature for predicting the prognosis among individuals with ccRCC^47^. However, the molecular mechanisms of the remaining four lncRNAs in various tumor types remain insufficiently explored. The discovery of these disulfidptosis-related lncRNAs represents a significant advancement in our understanding of ccRCC biology and provides new therapeutic targets. The accuracy and validity of the model were evaluated by dividing patients as per the median risk score into low- and high-risk groups. Validation analysis using the testing set and the entire set reinforced the accuracy of the model. Additionally, multivariate analyses exhibited that the risk model was capable of independently predicting the survival of the individuals. ROC and C-index curves evaluated the accuracy of the risk score regarding prognosis, revealing that this factor held the potential to act as a predictor of prognosis. The OS of patients was predicted through a nomogram. Additionally, calibration curves depicted high congruence between the actual/observed results and predictions. Moreover, the PCA of the constructed risk model revealed its ability to distinguish low- and high-risk individuals, which also implied that the risk model was a significant prognosis-predictive factor for individuals with ccRCC.

Furthermore, the investigation of the biological functions linked to the risk model based on the genes depicting differential expression between the two risk groups was executed. GO, KEGG, and GSEA revealed that immune function and immune-related pathways were significantly linked to disulfidptosis-related lncRNAs, indicating that the risk signature was not only linked to traditional cancer-related pathways but also to immune response. Following this, we investigated differences in the tumor immune microenvironment among different risk categories, identifying variations in immune-related functions and cells between high-risk and low-risk cohorts. Typically, rapidly growing tumors induce the development of a highly immunosuppressive TME that hampers the anti-tumor response and facilitates tumor invasion and progression^48^. However, recent studies have indicated a significant correlation between disulfidptosis and immune infiltration, wherein subtypes with high disulfidptosis levels demonstrate elevated immune scores^49^. Our findings align with these observations. Particularly, we identified increased expression levels of crucial anti-tumor immune cells, including CD8 + T cells, macrophages, and Th1 cells, within the high-risk group. Concurrently, the GSEA based on the C7 gene set identified an enrichment of up-regulated CD8 + T cells within the high-risk group. Our findings challenge the conventional belief that a high degree of CD8 + T cell infiltration universally indicates a favorable prognosis for survival. Additionally, several studies have reported that elevated levels of CD8 + T cells are sometimes associated with shorter survival times^50^. Moreover, within the high-risk group, there was a notable increase in Treg cells, which are key cells that inhibit anti-tumor immune responses and promote immune evasion^51^. This elevation of Treg cells could potentially contribute to the unfavorable prognosis observed in this particular population. Besides, the stromal, immune, and ESTIMATE scores in the different subgroups were assessed and it was observed that the high-risk groups had greater immune scores and lower tumor purity. These findings suggest that tumor immunosuppression might be high in the groups with high risk, which is indicative of a worse prognosis. Additionally, differences in immune escape and immunotherapy were assessed between the two risk groups. Greater TIDE scores were recorded for the group with high risk in contrast with groups with low risk. This was indicative of the increased possibility of immune evasion in the high-risk group along with the worsening of the immunotherapy effect. Based on these findings, a model-based sensitivity analysis of individuals with ccRCC to anti-cancer drugs was conducted, and the data found that axitinib had lower IC50 and higher sensitivity in the low-risk group, while savolitinib depicted enhanced efficacy in the high-risk group. Axitinib has been approved for treating advanced ccRCC after the failure of one prior systemic therapy^52^. Furthermore, savolitinib has demonstrated encouraging efficacy in individuals with MET-driven papillary renal cell carcinoma in Phase 3 randomized clinical trial compared to sunitinib^53^. However, reports on the use of sarvotinib for ccRCC treatments are scarce, requiring further cell-line drug sensitivity experiments. These findings could potentially guide clinicians to make personalized treatment decisions for different risk groups.

Despite the novel and promising findings, this study has certain limitations. Firstly, we only validated the results in the testing set of TCGA. Therefore, additional data from other databases are needed to verify the risk model. Secondly, further biological experiments are needed to elucidate the functions of hub disulfidptosis-related lncRNAs in ccRCC.

## Conclusion

This research is the first attempt made at establishing a disulfidptosis-related lncRNAs-based signature for ccRCC. By establishing this prognostic signature, the study presents a new measure for predicting outcomes among ccRCC individuals. The risk model demonstrated high accuracy in assessing patient survival outcomes and distinguishing between high and low-risk groups. Moreover, as per the remarkable variations in characteristics, such as immune infiltration, TMB, TIDE, and sensitivity to drugs, between the risk groups, this model has the potential to guide clinical decision-making for ccRCC treatment.

### Supplementary Information

Supplementary Table S1.

Supplementary Table S2.

Supplementary Figure S1.

Supplementary Figure S2.

## Data Availability

The datasets analyzed during the current study are available in The Cancer Genome Atlas (https://portal.gdc.cancer.gov). The original contributions presented in the study are included in the article/supplementary material, further inquiries can be directed to the corresponding authors.
