# Supplementary material for: Development of a novel disulfidptosis-related lncRNA signature for prognostic and immune response prediction in clear cell renal cell carcinoma
Source: Sci Rep. 2024 Jan 5;14:624. doi: 10.1038/s41598-024-51197-2 (PMC10770353; doi:10.1038/s41598-024-51197-2)
Supplement: Supplementary file 1 — Supplementary Table S1. [file 41598_2024_51197_MOESM1_ESM.docx]

**Supplementary Table S1** IncRNAs associated with each of the disulfidptosis gene.

| Disulfidptosis | LncRNA | Cor | adj*-P*-value | Regulation |
| --- | --- | --- | --- | --- |
| GYS1 | AC114803.1 | 0.485004 | 2.01E-28 | postive |
| GYS1 | LINC01426 | 0.435617 | 1.28E-21 | postive |
| GYS1 | LINC01522 | 0.424689 | 2.94E-20 | postive |
| GYS1 | LINC02188 | 0.453126 | 6.62E-24 | postive |
| GYS1 | PINK1-AS | 0.448953 | 2.39E-23 | postive |
| LRPPRC | AC002401.2 | 0.480775 | 8.5E-28 | postive |
| LRPPRC | AC003086.1 | 0.511339 | 1.56E-32 | postive |
| LRPPRC | AC004112.1 | 0.504609 | 1.9E-31 | postive |
| LRPPRC | AC004554.1 | 0.445013 | 7.89E-23 | postive |
| LRPPRC | AC005034.5 | 0.576513 | 2.14E-44 | postive |
| LRPPRC | AC005332.6 | 0.456502 | 2.32E-24 | postive |
| LRPPRC | AC005670.3 | 0.641356 | 3.55E-59 | postive |
| LRPPRC | AC006213.1 | 0.476203 | 3.96E-27 | postive |
| LRPPRC | AC006994.1 | 0.432012 | 3.65E-21 | postive |
| LRPPRC | AC007365.1 | 0.412606 | 8.26E-19 | postive |
| LRPPRC | AC007743.1 | 0.420388 | 9.79E-20 | postive |
| LRPPRC | AC008555.1 | 0.465878 | 1.18E-25 | postive |
| LRPPRC | AC008966.1 | 0.494734 | 6.7E-30 | postive |
| LRPPRC | AC011477.2 | 0.51137 | 1.54E-32 | postive |
| LRPPRC | AC011912.1 | 0.597138 | 9.81E-49 | postive |
| LRPPRC | AC015922.2 | 0.482053 | 5.51E-28 | postive |
| LRPPRC | AC016727.1 | 0.447978 | 3.21E-23 | postive |
| LRPPRC | AC018647.2 | 0.590688 | 2.41E-47 | postive |
| LRPPRC | AC018752.1 | 0.440707 | 2.86E-22 | postive |
| LRPPRC | AC021037.1 | 0.428879 | 8.97E-21 | postive |
| LRPPRC | AC021218.1 | 0.404566 | 7.05E-18 | postive |
| LRPPRC | AC026748.7 | 0.426446 | 1.79E-20 | postive |
| LRPPRC | AC034139.1 | 0.489149 | 4.78E-29 | postive |
| LRPPRC | AC064807.1 | 0.501984 | 4.95E-31 | postive |
| LRPPRC | AC073073.2 | 0.484338 | 2.52E-28 | postive |
| LRPPRC | AC073254.1 | 0.439557 | 4.02E-22 | postive |
| LRPPRC | AC079848.1 | 0.404521 | 7.13E-18 | postive |
| LRPPRC | AC084024.4 | -0.42569 | 2.22E-20 | negative |
| LRPPRC | AC091978.1 | 0.427528 | 1.32E-20 | postive |
| LRPPRC | AC091982.3 | 0.410272 | 1.55E-18 | postive |
| LRPPRC | AC092667.1 | 0.415364 | 3.9E-19 | postive |
| LRPPRC | AC093297.2 | 0.41599 | 3.29E-19 | postive |
| LRPPRC | AC095055.1 | 0.416488 | 2.87E-19 | postive |
| LRPPRC | AC104596.1 | 0.544169 | 3.49E-38 | postive |
| LRPPRC | AC107027.3 | 0.55887 | 6.35E-41 | postive |
| LRPPRC | AC108673.3 | -0.44885 | 2.47E-23 | negative |
| LRPPRC | AC112220.2 | 0.450818 | 1.35E-23 | postive |
| LRPPRC | AC120114.1 | 0.493487 | 1.04E-29 | postive |
| LRPPRC | AC121338.2 | 0.483761 | 3.07E-28 | postive |
| LRPPRC | AC124854.1 | 0.443808 | 1.13E-22 | postive |
| LRPPRC | ACVR2B-AS1 | 0.468674 | 4.75E-26 | postive |
| LRPPRC | AL022069.3 | 0.445226 | 7.4E-23 | postive |
| LRPPRC | AL035071.1 | -0.42921 | 8.15E-21 | negative |
| LRPPRC | AL078581.2 | 0.406444 | 4.3E-18 | postive |
| LRPPRC | AL078644.1 | 0.471521 | 1.87E-26 | postive |
| LRPPRC | AL132800.1 | 0.542765 | 6.26E-38 | postive |
| LRPPRC | AL137003.1 | 0.450769 | 1.37E-23 | postive |
| LRPPRC | AL161782.1 | 0.522931 | 1.86E-34 | postive |
| LRPPRC | AL162171.1 | 0.427223 | 1.44E-20 | postive |
| LRPPRC | AL353748.3 | 0.400145 | 2.24E-17 | postive |
| LRPPRC | AL450326.1 | 0.538844 | 3.17E-37 | postive |
| LRPPRC | AL590428.1 | 0.43046 | 5.7E-21 | postive |
| LRPPRC | AL592295.6 | 0.443184 | 1.37E-22 | postive |
| LRPPRC | AL606489.1 | 0.454301 | 4.6E-24 | postive |
| LRPPRC | AL732509.1 | 0.469896 | 3.19E-26 | postive |
| LRPPRC | AP000759.1 | 0.451546 | 1.08E-23 | postive |
| LRPPRC | AP001267.3 | 0.408267 | 2.65E-18 | postive |
| LRPPRC | AP001372.2 | 0.560618 | 2.94E-41 | postive |
| LRPPRC | AP001542.3 | 0.437458 | 7.47E-22 | postive |
| LRPPRC | AP003721.3 | 0.501511 | 5.87E-31 | postive |
| LRPPRC | AP003721.4 | 0.432844 | 2.87E-21 | postive |
| LRPPRC | ATXN1-AS1 | 0.470193 | 2.89E-26 | postive |
| LRPPRC | BAIAP2-DT | 0.417867 | 1.97E-19 | postive |
| LRPPRC | CCNT2-AS1 | 0.484088 | 2.75E-28 | postive |
| LRPPRC | CTBP1-DT | 0.508598 | 4.35E-32 | postive |
| LRPPRC | DHRS4-AS1 | 0.463696 | 2.37E-25 | postive |
| LRPPRC | EDRF1-DT | 0.425995 | 2.04E-20 | postive |
| LRPPRC | EIF2AK3-DT | 0.457069 | 1.94E-24 | postive |
| LRPPRC | EIF3J-DT | 0.493897 | 9.01E-30 | postive |
| LRPPRC | EMX2OS | 0.510844 | 1.88E-32 | postive |
| LRPPRC | EPB41L4A-AS1 | 0.404278 | 7.61E-18 | postive |
| LRPPRC | FAM111A-DT | 0.468196 | 5.55E-26 | postive |
| LRPPRC | FAM160A1-DT | 0.458566 | 1.21E-24 | postive |
| LRPPRC | FGD5-AS1 | 0.456498 | 2.32E-24 | postive |
| LRPPRC | FZD4-DT | 0.490921 | 2.57E-29 | postive |
| LRPPRC | GNG12-AS1 | 0.428613 | 9.68E-21 | postive |
| LRPPRC | HAGLR | 0.412392 | 8.75E-19 | postive |
| LRPPRC | HCG15 | 0.44279 | 1.54E-22 | postive |
| LRPPRC | HMGN3-AS1 | 0.413837 | 5.91E-19 | postive |
| LRPPRC | LINC00863 | 0.434046 | 2.02E-21 | postive |
| LRPPRC | LINC01521 | 0.438537 | 5.44E-22 | postive |
| LRPPRC | LINC01534 | 0.416779 | 2.65E-19 | postive |
| LRPPRC | LINC01550 | 0.41498 | 4.33E-19 | postive |
| LRPPRC | LINC01671 | 0.458186 | 1.37E-24 | postive |
| LRPPRC | LINC01801 | 0.495375 | 5.33E-30 | postive |
| LRPPRC | LINC01963 | 0.433731 | 2.22E-21 | postive |
| LRPPRC | LINCMD1 | 0.416385 | 2.95E-19 | postive |
| LRPPRC | MALINC1 | 0.428044 | 1.14E-20 | postive |
| LRPPRC | MAP4K3-DT | 0.438017 | 6.34E-22 | postive |
| LRPPRC | MKLN1-AS | 0.495563 | 4.99E-30 | postive |
| LRPPRC | MRPS30-DT | 0.412128 | 9.4E-19 | postive |
| LRPPRC | NIFK-AS1 | 0.450068 | 1.7E-23 | postive |
| LRPPRC | NNT-AS1 | 0.561962 | 1.62E-41 | postive |
| LRPPRC | NRAV | 0.441131 | 2.52E-22 | postive |
| LRPPRC | NRSN2-AS1 | 0.555205 | 3.15E-40 | postive |
| LRPPRC | OTUD6B-AS1 | 0.565072 | 4.03E-42 | postive |
| LRPPRC | PAXIP1-AS2 | 0.568344 | 9.2E-43 | postive |
| LRPPRC | RAP2C-AS1 | 0.539396 | 2.53E-37 | postive |
| LRPPRC | RBM26-AS1 | 0.400411 | 2.09E-17 | postive |
| LRPPRC | SBF2-AS1 | 0.403647 | 8.98E-18 | postive |
| LRPPRC | SEPTIN7-DT | 0.536783 | 7.38E-37 | postive |
| LRPPRC | SGMS1-AS1 | 0.570531 | 3.39E-43 | postive |
| LRPPRC | SMARCA5-AS1 | 0.531746 | 5.67E-36 | postive |
| LRPPRC | SNHG16 | 0.579633 | 4.93E-45 | postive |
| LRPPRC | SNHG29 | 0.511032 | 1.75E-32 | postive |
| LRPPRC | SNHG8 | 0.404954 | 6.37E-18 | postive |
| LRPPRC | SP2-AS1 | 0.473973 | 8.32E-27 | postive |
| LRPPRC | STK4-AS1 | 0.537569 | 5.35E-37 | postive |
| LRPPRC | SUCLG2-AS1 | 0.412798 | 7.84E-19 | postive |
| LRPPRC | TBC1D8-AS1 | 0.485558 | 1.66E-28 | postive |
| LRPPRC | TNFRSF10A-AS1 | 0.405791 | 5.11E-18 | postive |
| LRPPRC | TRAM2-AS1 | 0.614345 | 1.33E-52 | postive |
| LRPPRC | U91328.1 | 0.591638 | 1.51E-47 | postive |
| LRPPRC | UBL7-AS1 | 0.430985 | 4.9E-21 | postive |
| LRPPRC | UGDH-AS1 | 0.547349 | 9.14E-39 | postive |
| LRPPRC | USP27X-AS1 | 0.511167 | 1.67E-32 | postive |
| LRPPRC | USP46-DT | 0.515522 | 3.22E-33 | postive |
| LRPPRC | UXT-AS1 | 0.42965 | 7.19E-21 | postive |
| LRPPRC | WASL-DT | 0.553139 | 7.71E-40 | postive |
| LRPPRC | WDFY3-AS2 | 0.508416 | 4.65E-32 | postive |
| LRPPRC | ZNF22-AS1 | 0.42496 | 2.73E-20 | postive |
| NCKAP1 | AC002401.2 | 0.400664 | 1.96E-17 | postive |
| NCKAP1 | AC003086.1 | 0.405837 | 5.04E-18 | postive |
| NCKAP1 | AC004112.1 | 0.521235 | 3.59E-34 | postive |
| NCKAP1 | AC004554.1 | 0.535216 | 1.4E-36 | postive |
| NCKAP1 | AC005034.5 | 0.617168 | 2.93E-53 | postive |
| NCKAP1 | AC005332.6 | 0.608605 | 2.76E-51 | postive |
| NCKAP1 | AC005670.3 | 0.598641 | 4.61E-49 | postive |
| NCKAP1 | AC006213.1 | 0.475164 | 5.6E-27 | postive |
| NCKAP1 | AC007365.1 | 0.421674 | 6.85E-20 | postive |
| NCKAP1 | AC007405.3 | 0.401748 | 1.48E-17 | postive |
| NCKAP1 | AC007485.1 | 0.407492 | 3.25E-18 | postive |
| NCKAP1 | AC007743.1 | 0.463464 | 2.56E-25 | postive |
| NCKAP1 | AC008124.1 | 0.401194 | 1.7E-17 | postive |
| NCKAP1 | AC008555.1 | 0.487151 | 9.57E-29 | postive |
| NCKAP1 | AC009318.2 | 0.470209 | 2.88E-26 | postive |
| NCKAP1 | AC009318.3 | 0.406002 | 4.83E-18 | postive |
| NCKAP1 | AC009962.1 | 0.464195 | 2.02E-25 | postive |
| NCKAP1 | AC011477.2 | 0.578661 | 7.8E-45 | postive |
| NCKAP1 | AC011912.1 | 0.559534 | 4.74E-41 | postive |
| NCKAP1 | AC012306.2 | 0.435397 | 1.37E-21 | postive |
| NCKAP1 | AC015922.2 | 0.597935 | 6.57E-49 | postive |
| NCKAP1 | AC015922.3 | 0.437003 | 8.54E-22 | postive |
| NCKAP1 | AC016727.1 | 0.41323 | 6.97E-19 | postive |
| NCKAP1 | AC017099.2 | 0.44872 | 2.56E-23 | postive |
| NCKAP1 | AC018645.3 | 0.407931 | 2.89E-18 | postive |
| NCKAP1 | AC018647.2 | 0.612694 | 3.21E-52 | postive |
| NCKAP1 | AC018752.1 | 0.420565 | 9.32E-20 | postive |
| NCKAP1 | AC021037.1 | 0.42045 | 9.63E-20 | postive |
| NCKAP1 | AC034139.1 | 0.507492 | 6.55E-32 | postive |
| NCKAP1 | AC064807.1 | 0.509969 | 2.61E-32 | postive |
| NCKAP1 | AC068870.2 | 0.450194 | 1.63E-23 | postive |
| NCKAP1 | AC073073.2 | 0.582474 | 1.28E-45 | postive |
| NCKAP1 | AC073254.1 | 0.485516 | 1.68E-28 | postive |
| NCKAP1 | AC083799.1 | 0.41417 | 5.4E-19 | postive |
| NCKAP1 | AC090198.1 | 0.443874 | 1.11E-22 | postive |
| NCKAP1 | AC091563.1 | 0.416846 | 2.6E-19 | postive |
| NCKAP1 | AC095055.1 | 0.429699 | 7.09E-21 | postive |
| NCKAP1 | AC096921.2 | 0.512171 | 1.14E-32 | postive |
| NCKAP1 | AC097639.1 | 0.41095 | 1.29E-18 | postive |
| NCKAP1 | AC103591.4 | 0.460682 | 6.2E-25 | postive |
| NCKAP1 | AC104596.1 | 0.443162 | 1.38E-22 | postive |
| NCKAP1 | AC107027.3 | 0.655117 | 8.68E-63 | postive |
| NCKAP1 | AC108047.1 | 0.438491 | 5.51E-22 | postive |
| NCKAP1 | AC108463.2 | 0.442458 | 1.7E-22 | postive |
| NCKAP1 | AC108673.3 | -0.41278 | 7.87E-19 | negative |
| NCKAP1 | AC112220.2 | 0.467643 | 6.64E-26 | postive |
| NCKAP1 | AC120114.1 | 0.402874 | 1.1E-17 | postive |
| NCKAP1 | AC244517.7 | 0.409588 | 1.86E-18 | postive |
| NCKAP1 | AF127577.4 | 0.539981 | 1.99E-37 | postive |
| NCKAP1 | AF241728.2 | 0.402831 | 1.11E-17 | postive |
| NCKAP1 | AL022069.3 | 0.42126 | 7.68E-20 | postive |
| NCKAP1 | AL078581.2 | 0.500657 | 8.01E-31 | postive |
| NCKAP1 | AL078644.1 | 0.414422 | 5.05E-19 | postive |
| NCKAP1 | AL121603.2 | 0.471247 | 2.05E-26 | postive |
| NCKAP1 | AL132800.1 | 0.509936 | 2.64E-32 | postive |
| NCKAP1 | AL135925.1 | 0.468106 | 5.71E-26 | postive |
| NCKAP1 | AL137003.1 | 0.434917 | 1.57E-21 | postive |
| NCKAP1 | AL158206.1 | 0.401943 | 1.4E-17 | postive |
| NCKAP1 | AL161782.1 | 0.454256 | 4.67E-24 | postive |
| NCKAP1 | AL450326.1 | 0.51079 | 1.92E-32 | postive |
| NCKAP1 | AL590428.1 | 0.423779 | 3.8E-20 | postive |
| NCKAP1 | AL592295.6 | 0.467736 | 6.44E-26 | postive |
| NCKAP1 | AL606489.1 | 0.431155 | 4.67E-21 | postive |
| NCKAP1 | AL732509.1 | 0.434035 | 2.03E-21 | postive |
| NCKAP1 | AP000759.1 | 0.493909 | 8.97E-30 | postive |
| NCKAP1 | AP001318.2 | 0.438042 | 6.29E-22 | postive |
| NCKAP1 | AP001372.2 | 0.661961 | 1.18E-64 | postive |
| NCKAP1 | AP001542.3 | 0.406665 | 4.05E-18 | postive |
| NCKAP1 | AP003721.3 | 0.560776 | 2.74E-41 | postive |
| NCKAP1 | BAIAP2-DT | 0.459903 | 7.94E-25 | postive |
| NCKAP1 | CARD8-AS1 | 0.443715 | 1.17E-22 | postive |
| NCKAP1 | CASC2 | 0.458475 | 1.25E-24 | postive |
| NCKAP1 | CCNT2-AS1 | 0.465165 | 1.48E-25 | postive |
| NCKAP1 | CLCA4-AS1 | 0.415469 | 3.79E-19 | postive |
| NCKAP1 | CTBP1-DT | 0.537886 | 4.7E-37 | postive |
| NCKAP1 | DHRS4-AS1 | 0.501398 | 6.12E-31 | postive |
| NCKAP1 | DNAJC3-DT | 0.417323 | 2.28E-19 | postive |
| NCKAP1 | EIF2AK3-DT | 0.437328 | 7.76E-22 | postive |
| NCKAP1 | EIF3J-DT | 0.440214 | 3.31E-22 | postive |
| NCKAP1 | EMX2OS | 0.501019 | 7.02E-31 | postive |
| NCKAP1 | FAM111A-DT | 0.47008 | 3E-26 | postive |
| NCKAP1 | FGD5-AS1 | 0.572249 | 1.54E-43 | postive |
| NCKAP1 | FZD4-DT | 0.41979 | 1.16E-19 | postive |
| NCKAP1 | GNG12-AS1 | 0.401579 | 1.54E-17 | postive |
| NCKAP1 | HAGLR | 0.420959 | 8.35E-20 | postive |
| NCKAP1 | HMGN3-AS1 | 0.432234 | 3.42E-21 | postive |
| NCKAP1 | LINC00667 | 0.427094 | 1.49E-20 | postive |
| NCKAP1 | LINC00863 | 0.539745 | 2.19E-37 | postive |
| NCKAP1 | LINC01415 | 0.472718 | 1.26E-26 | postive |
| NCKAP1 | LINC01521 | 0.429121 | 8.37E-21 | postive |
| NCKAP1 | LINC01671 | 0.416391 | 2.95E-19 | postive |
| NCKAP1 | LINC01801 | 0.459732 | 8.38E-25 | postive |
| NCKAP1 | LINC01963 | 0.466945 | 8.33E-26 | postive |
| NCKAP1 | LINC02027 | 0.417339 | 2.27E-19 | postive |
| NCKAP1 | MALINC1 | 0.429079 | 8.47E-21 | postive |
| NCKAP1 | MKLN1-AS | 0.496098 | 4.12E-30 | postive |
| NCKAP1 | MRPS30-DT | 0.420452 | 9.62E-20 | postive |
| NCKAP1 | MSC-AS1 | 0.508937 | 3.83E-32 | postive |
| NCKAP1 | NIFK-AS1 | 0.458447 | 1.26E-24 | postive |
| NCKAP1 | NNT-AS1 | 0.481775 | 6.05E-28 | postive |
| NCKAP1 | NRAV | 0.434493 | 1.78E-21 | postive |
| NCKAP1 | NRSN2-AS1 | 0.418517 | 1.64E-19 | postive |
| NCKAP1 | NUTM2A-AS1 | 0.427687 | 1.26E-20 | postive |
| NCKAP1 | OTUD6B-AS1 | 0.664485 | 2.34E-65 | postive |
| NCKAP1 | PAXIP1-AS2 | 0.567636 | 1.27E-42 | postive |
| NCKAP1 | RAP2C-AS1 | 0.591335 | 1.75E-47 | postive |
| NCKAP1 | RBM26-AS1 | 0.424198 | 3.38E-20 | postive |
| NCKAP1 | SEPTIN7-DT | 0.435854 | 1.2E-21 | postive |
| NCKAP1 | SETBP1-DT | 0.402751 | 1.14E-17 | postive |
| NCKAP1 | SGMS1-AS1 | 0.546147 | 1.52E-38 | postive |
| NCKAP1 | SMARCA5-AS1 | 0.532646 | 3.95E-36 | postive |
| NCKAP1 | SNHG16 | 0.490045 | 3.49E-29 | postive |
| NCKAP1 | SP2-AS1 | 0.462321 | 3.68E-25 | postive |
| NCKAP1 | STK4-AS1 | 0.413851 | 5.89E-19 | postive |
| NCKAP1 | SUCLG2-AS1 | 0.449553 | 1.99E-23 | postive |
| NCKAP1 | TNFRSF10A-AS1 | 0.472959 | 1.16E-26 | postive |
| NCKAP1 | TRAM2-AS1 | 0.650067 | 1.93E-61 | postive |
| NCKAP1 | U91328.1 | 0.533224 | 3.13E-36 | postive |
| NCKAP1 | UBL7-AS1 | 0.429971 | 6.56E-21 | postive |
| NCKAP1 | UGDH-AS1 | 0.503679 | 2.67E-31 | postive |
| NCKAP1 | USP27X-AS1 | 0.492706 | 1.37E-29 | postive |
| NCKAP1 | USP46-DT | 0.633604 | 3.19E-57 | postive |
| NCKAP1 | WAC-AS1 | 0.43105 | 4.81E-21 | postive |
| NCKAP1 | WASL-DT | 0.537801 | 4.87E-37 | postive |
| NCKAP1 | WDFY3-AS2 | 0.591946 | 1.3E-47 | postive |
| NCKAP1 | ZNF22-AS1 | 0.46798 | 5.95E-26 | postive |
| NDUFA11 | AC004080.4 | 0.406905 | 3.8E-18 | postive |
| NDUFA11 | AC005498.2 | 0.511584 | 1.42E-32 | postive |
| NDUFA11 | AC006538.1 | 0.408492 | 2.49E-18 | postive |
| NDUFA11 | AC008622.2 | 0.483754 | 3.08E-28 | postive |
| NDUFA11 | AC008897.3 | 0.435555 | 1.3E-21 | postive |
| NDUFA11 | AC008915.2 | 0.423891 | 3.68E-20 | postive |
| NDUFA11 | AC009090.1 | 0.439623 | 3.95E-22 | postive |
| NDUFA11 | AC009309.1 | 0.797162 | 2.3E-115 | postive |
| NDUFA11 | AC009554.2 | 0.523404 | 1.54E-34 | postive |
| NDUFA11 | AC010913.1 | 0.524629 | 9.55E-35 | postive |
| NDUFA11 | AC012065.2 | 0.460775 | 6.02E-25 | postive |
| NDUFA11 | AC015802.5 | 0.51858 | 9.99E-34 | postive |
| NDUFA11 | AC018529.1 | 0.434062 | 2.02E-21 | postive |
| NDUFA11 | AC023509.3 | 0.651811 | 6.66E-62 | postive |
| NDUFA11 | AC026803.2 | 0.430315 | 5.95E-21 | postive |
| NDUFA11 | AC034102.3 | 0.59793 | 6.59E-49 | postive |
| NDUFA11 | AC040169.1 | 0.489777 | 3.84E-29 | postive |
| NDUFA11 | AC046143.2 | 0.411444 | 1.13E-18 | postive |
| NDUFA11 | AC067852.2 | 0.733606 | 1.23E-87 | postive |
| NDUFA11 | AC068338.3 | 0.416209 | 3.1E-19 | postive |
| NDUFA11 | AC068492.1 | 0.476504 | 3.58E-27 | postive |
| NDUFA11 | AC087379.2 | 0.570119 | 4.1E-43 | postive |
| NDUFA11 | AC087623.2 | 0.4174 | 2.23E-19 | postive |
| NDUFA11 | AC092119.3 | 0.467269 | 7.5E-26 | postive |
| NDUFA11 | AC092306.1 | 0.480284 | 1E-27 | postive |
| NDUFA11 | AC099518.6 | 0.646703 | 1.47E-60 | postive |
| NDUFA11 | AC099791.2 | 0.414888 | 4.44E-19 | postive |
| NDUFA11 | AC103724.4 | 0.433491 | 2.38E-21 | postive |
| NDUFA11 | AC104316.2 | 0.496663 | 3.37E-30 | postive |
| NDUFA11 | AC130371.2 | 0.446439 | 5.13E-23 | postive |
| NDUFA11 | AC131009.3 | 0.451942 | 9.55E-24 | postive |
| NDUFA11 | AC133552.5 | 0.61368 | 1.9E-52 | postive |
| NDUFA11 | AL021707.6 | 0.461654 | 4.55E-25 | postive |
| NDUFA11 | AL023803.1 | 0.907973 | 6.5E-201 | postive |
| NDUFA11 | AL031733.2 | 0.556927 | 1.49E-40 | postive |
| NDUFA11 | AL035587.2 | 0.501197 | 6.58E-31 | postive |
| NDUFA11 | AL121832.2 | 0.573899 | 7.2E-44 | postive |
| NDUFA11 | AL121899.1 | 0.691989 | 1.82E-73 | postive |
| NDUFA11 | AL121944.1 | 0.749808 | 6.67E-94 | postive |
| NDUFA11 | AL138966.2 | 0.427559 | 1.31E-20 | postive |
| NDUFA11 | AL161729.3 | 0.830739 | 2.2E-134 | postive |
| NDUFA11 | AL354953.1 | 0.574924 | 4.48E-44 | postive |
| NDUFA11 | AL355353.1 | 0.629584 | 3.13E-56 | postive |
| NDUFA11 | AL359878.2 | 0.430584 | 5.5E-21 | postive |
| NDUFA11 | AL391261.2 | 0.513728 | 6.35E-33 | postive |
| NDUFA11 | AL391883.1 | 0.469416 | 3.73E-26 | postive |
| NDUFA11 | AL513165.1 | 0.712894 | 2.87E-80 | postive |
| NDUFA11 | ANK3-DT | 0.419466 | 1.26E-19 | postive |
| NDUFA11 | AP000696.2 | 0.465483 | 1.34E-25 | postive |
| NDUFA11 | AP001207.3 | 0.404863 | 6.52E-18 | postive |
| NDUFA11 | AP001363.2 | 0.732181 | 4.15E-87 | postive |
| NDUFA11 | AP001505.1 | 0.654024 | 1.71E-62 | postive |
| NDUFA11 | AP003032.1 | 0.752821 | 4.03E-95 | postive |
| NDUFA11 | AP003307.1 | 0.445723 | 6.37E-23 | postive |
| NDUFA11 | AP007216.2 | 0.616905 | 3.38E-53 | postive |
| NDUFA11 | BNC2-AS1 | 0.419345 | 1.31E-19 | postive |
| NDUFA11 | BX649632.1 | 0.404655 | 6.89E-18 | postive |
| NDUFA11 | CAMTA1-DT | 0.558837 | 6.44E-41 | postive |
| NDUFA11 | CDC37L1-DT | 0.542513 | 6.96E-38 | postive |
| NDUFA11 | CH17-340M24.3 | 0.874964 | 4.6E-167 | postive |
| NDUFA11 | CYTOR | 0.426682 | 1.68E-20 | postive |
| NDUFA11 | ENTPD3-AS1 | 0.667351 | 3.68E-66 | postive |
| NDUFA11 | FARSA-AS1 | 0.449984 | 1.74E-23 | postive |
| NDUFA11 | FOXC2-AS1 | 0.877982 | 9.7E-170 | postive |
| NDUFA11 | GATA3-AS1 | 0.844515 | 1.9E-143 | postive |
| NDUFA11 | HEIH | 0.512698 | 9.37E-33 | postive |
| NDUFA11 | HSD11B1-AS1 | 0.802493 | 3.9E-118 | postive |
| NDUFA11 | LAMA5-AS1 | 0.531836 | 5.47E-36 | postive |
| NDUFA11 | LINC01023 | 0.891897 | 4.7E-183 | postive |
| NDUFA11 | LINC01976 | 0.738796 | 1.36E-89 | postive |
| NDUFA11 | LINC01983 | 0.927961 | 2.3E-228 | postive |
| NDUFA11 | LIPE-AS1 | 0.495023 | 6.04E-30 | postive |
| NDUFA11 | PRDM16-DT | 0.416848 | 2.6E-19 | postive |
| NDUFA11 | PRR34-AS1 | 0.66075 | 2.54E-64 | postive |
| NDUFA11 | RAB11B-AS1 | 0.444562 | 9.04E-23 | postive |
| NDUFA11 | RNF207-AS1 | 0.658854 | 8.41E-64 | postive |
| NDUFA11 | RPARP-AS1 | 0.783285 | 1.6E-108 | postive |
| NDUFA11 | SEMA3B-AS1 | 0.431951 | 3.71E-21 | postive |
| NDUFA11 | SNAI3-AS1 | 0.40458 | 7.02E-18 | postive |
| NDUFA11 | SNHG19 | 0.895865 | 3.5E-187 | postive |
| NDUFA11 | SNHG25 | 0.500216 | 9.39E-31 | postive |
| NDUFA11 | SNHG9 | 0.530409 | 9.69E-36 | postive |
| NDUFA11 | SPINT1-AS1 | 0.460013 | 7.67E-25 | postive |
| NDUFA11 | SUCLA2-AS1 | 0.576202 | 2.47E-44 | postive |
| NDUFA11 | TBX2-AS1 | 0.481935 | 5.73E-28 | postive |
| NDUFA11 | Z97192.2 | 0.404946 | 6.38E-18 | postive |
| NDUFA11 | Z97653.1 | 0.621453 | 2.86E-54 | postive |
| NDUFA11 | ZSCAN16-AS1 | 0.879971 | 1.5E-171 | postive |
| NDUFS1 | AC002401.2 | 0.432042 | 3.62E-21 | postive |
| NDUFS1 | AC004112.1 | 0.515235 | 3.59E-33 | postive |
| NDUFS1 | AC004554.1 | 0.408174 | 2.71E-18 | postive |
| NDUFS1 | AC005034.5 | 0.458644 | 1.18E-24 | postive |
| NDUFS1 | AC005670.3 | 0.555888 | 2.34E-40 | postive |
| NDUFS1 | AC007637.1 | 0.459248 | 9.77E-25 | postive |
| NDUFS1 | AC008124.1 | 0.471951 | 1.62E-26 | postive |
| NDUFS1 | AC011477.2 | 0.441131 | 2.52E-22 | postive |
| NDUFS1 | AC012313.5 | 0.405259 | 5.87E-18 | postive |
| NDUFS1 | AC015922.2 | 0.539488 | 2.43E-37 | postive |
| NDUFS1 | AC017100.1 | 0.461471 | 4.83E-25 | postive |
| NDUFS1 | AC018647.2 | 0.480214 | 1.03E-27 | postive |
| NDUFS1 | AC021037.1 | 0.402104 | 1.34E-17 | postive |
| NDUFS1 | AC026691.1 | 0.41561 | 3.65E-19 | postive |
| NDUFS1 | AC026992.2 | 0.412624 | 8.22E-19 | postive |
| NDUFS1 | AC064807.1 | 0.437554 | 7.26E-22 | postive |
| NDUFS1 | AC068338.2 | 0.479678 | 1.23E-27 | postive |
| NDUFS1 | AC073254.1 | 0.561134 | 2.34E-41 | postive |
| NDUFS1 | AC079848.1 | 0.509588 | 3.01E-32 | postive |
| NDUFS1 | AC092295.2 | 0.44063 | 2.93E-22 | postive |
| NDUFS1 | AC092296.1 | 0.407276 | 3.44E-18 | postive |
| NDUFS1 | AC097359.2 | 0.560879 | 2.62E-41 | postive |
| NDUFS1 | AC104109.2 | 0.507329 | 6.96E-32 | postive |
| NDUFS1 | AC106791.1 | 0.428092 | 1.12E-20 | postive |
| NDUFS1 | AC107027.3 | 0.545917 | 1.67E-38 | postive |
| NDUFS1 | AC108693.2 | 0.419235 | 1.35E-19 | postive |
| NDUFS1 | AC112220.2 | 0.516886 | 1.91E-33 | postive |
| NDUFS1 | AF111167.2 | 0.412176 | 9.27E-19 | postive |
| NDUFS1 | AL023806.1 | 0.426257 | 1.89E-20 | postive |
| NDUFS1 | AL035411.3 | 0.407603 | 3.16E-18 | postive |
| NDUFS1 | AL078581.2 | 0.537126 | 6.42E-37 | postive |
| NDUFS1 | AL136040.1 | 0.407567 | 3.19E-18 | postive |
| NDUFS1 | AL161782.1 | 0.49441 | 7.51E-30 | postive |
| NDUFS1 | AL162377.1 | 0.412841 | 7.75E-19 | postive |
| NDUFS1 | AL353748.3 | 0.537563 | 5.37E-37 | postive |
| NDUFS1 | AL359704.2 | 0.444116 | 1.03E-22 | postive |
| NDUFS1 | AL449106.1 | 0.422436 | 5.54E-20 | postive |
| NDUFS1 | AL450326.1 | 0.561158 | 2.31E-41 | postive |
| NDUFS1 | AL512603.2 | 0.402593 | 1.18E-17 | postive |
| NDUFS1 | AL592295.6 | 0.480059 | 1.08E-27 | postive |
| NDUFS1 | AL606489.1 | 0.507751 | 5.95E-32 | postive |
| NDUFS1 | AP001542.3 | 0.43577 | 1.23E-21 | postive |
| NDUFS1 | AP003721.3 | 0.420951 | 8.37E-20 | postive |
| NDUFS1 | CCDC183-AS1 | 0.501431 | 6.05E-31 | postive |
| NDUFS1 | DHRS4-AS1 | 0.523228 | 1.65E-34 | postive |
| NDUFS1 | DNAJC3-DT | 0.570487 | 3.46E-43 | postive |
| NDUFS1 | EIF2AK3-DT | 0.438092 | 6.2E-22 | postive |
| NDUFS1 | EMX2OS | 0.506618 | 9.05E-32 | postive |
| NDUFS1 | FGD5-AS1 | 0.489035 | 4.97E-29 | postive |
| NDUFS1 | GAS5-AS1 | 0.45173 | 1.02E-23 | postive |
| NDUFS1 | HMGN3-AS1 | 0.416764 | 2.66E-19 | postive |
| NDUFS1 | LINC00571 | 0.409888 | 1.72E-18 | postive |
| NDUFS1 | LINC00863 | 0.416114 | 3.18E-19 | postive |
| NDUFS1 | LINC01521 | 0.485599 | 1.63E-28 | postive |
| NDUFS1 | LINC01852 | 0.408854 | 2.26E-18 | postive |
| NDUFS1 | LINC02027 | 0.413364 | 6.73E-19 | postive |
| NDUFS1 | MAP4K3-DT | 0.449967 | 1.75E-23 | postive |
| NDUFS1 | NNT-AS1 | 0.547216 | 9.68E-39 | postive |
| NDUFS1 | NRAV | 0.403478 | 9.38E-18 | postive |
| NDUFS1 | OTUD6B-AS1 | 0.532729 | 3.82E-36 | postive |
| NDUFS1 | PAXIP1-AS2 | 0.501917 | 5.07E-31 | postive |
| NDUFS1 | PCAT7 | 0.493665 | 9.78E-30 | postive |
| NDUFS1 | PLBD1-AS1 | 0.449259 | 2.18E-23 | postive |
| NDUFS1 | PPIC-AS1 | 0.436168 | 1.09E-21 | postive |
| NDUFS1 | PTOV1-AS1 | 0.401709 | 1.49E-17 | postive |
| NDUFS1 | RAP2C-AS1 | 0.496212 | 3.96E-30 | postive |
| NDUFS1 | SGMS1-AS1 | 0.44067 | 2.89E-22 | postive |
| NDUFS1 | SLC25A5-AS1 | 0.500674 | 7.96E-31 | postive |
| NDUFS1 | SMARCA5-AS1 | 0.471581 | 1.83E-26 | postive |
| NDUFS1 | SUCLG2-AS1 | 0.565951 | 2.72E-42 | postive |
| NDUFS1 | TAF1A-AS1 | 0.401712 | 1.49E-17 | postive |
| NDUFS1 | U91328.1 | 0.612625 | 3.33E-52 | postive |
| NDUFS1 | USP46-DT | 0.60911 | 2.12E-51 | postive |
| NDUFS1 | WASL-DT | 0.471149 | 2.11E-26 | postive |
| NDUFS1 | WDFY3-AS2 | 0.507983 | 5.46E-32 | postive |
| NDUFS1 | ZNF710-AS1 | 0.405572 | 5.41E-18 | postive |
| NUBPL | AC004112.1 | 0.463881 | 2.24E-25 | postive |
| NUBPL | AC004554.1 | 0.509814 | 2.76E-32 | postive |
| NUBPL | AC004918.3 | 0.42222 | 5.88E-20 | postive |
| NUBPL | AC005034.5 | 0.563654 | 7.61E-42 | postive |
| NUBPL | AC005332.6 | 0.512007 | 1.22E-32 | postive |
| NUBPL | AC005498.3 | 0.507919 | 5.59E-32 | postive |
| NUBPL | AC005670.3 | 0.597 | 1.05E-48 | postive |
| NUBPL | AC006116.9 | 0.496877 | 3.12E-30 | postive |
| NUBPL | AC006213.1 | 0.512896 | 8.7E-33 | postive |
| NUBPL | AC006213.4 | 0.498432 | 1.79E-30 | postive |
| NUBPL | AC006994.1 | 0.446496 | 5.04E-23 | postive |
| NUBPL | AC007066.2 | 0.430207 | 6.13E-21 | postive |
| NUBPL | AC007365.1 | 0.416295 | 3.03E-19 | postive |
| NUBPL | AC007637.1 | 0.519054 | 8.33E-34 | postive |
| NUBPL | AC007743.1 | 0.512213 | 1.12E-32 | postive |
| NUBPL | AC008124.1 | 0.534196 | 2.11E-36 | postive |
| NUBPL | AC008494.2 | 0.405778 | 5.12E-18 | postive |
| NUBPL | AC008537.2 | 0.487118 | 9.67E-29 | postive |
| NUBPL | AC008543.1 | 0.435511 | 1.32E-21 | postive |
| NUBPL | AC008555.1 | 0.463364 | 2.64E-25 | postive |
| NUBPL | AC008937.3 | 0.418133 | 1.83E-19 | postive |
| NUBPL | AC009318.2 | 0.41434 | 5.16E-19 | postive |
| NUBPL | AC009486.1 | 0.44533 | 7.17E-23 | postive |
| NUBPL | AC009962.1 | 0.530062 | 1.11E-35 | postive |
| NUBPL | AC010615.2 | 0.420838 | 8.64E-20 | postive |
| NUBPL | AC011477.1 | 0.511871 | 1.28E-32 | postive |
| NUBPL | AC011477.2 | 0.589041 | 5.39E-47 | postive |
| NUBPL | AC011912.1 | 0.452933 | 7.03E-24 | postive |
| NUBPL | AC012313.1 | 0.479269 | 1.42E-27 | postive |
| NUBPL | AC012313.5 | 0.530525 | 9.25E-36 | postive |
| NUBPL | AC015922.2 | 0.462678 | 3.29E-25 | postive |
| NUBPL | AC016727.1 | 0.433729 | 2.22E-21 | postive |
| NUBPL | AC017099.2 | 0.429358 | 7.82E-21 | postive |
| NUBPL | AC018521.6 | 0.459735 | 8.37E-25 | postive |
| NUBPL | AC018647.2 | 0.576147 | 2.53E-44 | postive |
| NUBPL | AC018752.1 | 0.515219 | 3.61E-33 | postive |
| NUBPL | AC018809.2 | 0.475033 | 5.85E-27 | postive |
| NUBPL | AC020779.2 | 0.432898 | 2.82E-21 | postive |
| NUBPL | AC020915.2 | 0.405642 | 5.31E-18 | postive |
| NUBPL | AC021087.1 | 0.416962 | 2.52E-19 | postive |
| NUBPL | AC022211.2 | 0.405454 | 5.58E-18 | postive |
| NUBPL | AC026471.1 | 0.504052 | 2.33E-31 | postive |
| NUBPL | AC026979.4 | 0.425031 | 2.67E-20 | postive |
| NUBPL | AC034139.1 | 0.451251 | 1.18E-23 | postive |
| NUBPL | AC036214.2 | 0.471605 | 1.82E-26 | postive |
| NUBPL | AC046134.2 | 0.422855 | 4.92E-20 | postive |
| NUBPL | AC064807.1 | 0.545114 | 2.35E-38 | postive |
| NUBPL | AC068620.1 | 0.431763 | 3.92E-21 | postive |
| NUBPL | AC073073.2 | 0.506596 | 9.13E-32 | postive |
| NUBPL | AC078883.1 | 0.406288 | 4.48E-18 | postive |
| NUBPL | AC079142.1 | 0.438418 | 5.63E-22 | postive |
| NUBPL | AC087071.2 | 0.443036 | 1.43E-22 | postive |
| NUBPL | AC090198.1 | 0.536851 | 7.18E-37 | postive |
| NUBPL | AC092295.2 | 0.408548 | 2.46E-18 | postive |
| NUBPL | AC092329.4 | 0.405007 | 6.28E-18 | postive |
| NUBPL | AC092802.1 | 0.490728 | 2.75E-29 | postive |
| NUBPL | AC093227.3 | 0.441733 | 2.11E-22 | postive |
| NUBPL | AC093297.2 | 0.446585 | 4.91E-23 | postive |
| NUBPL | AC093510.1 | 0.430731 | 5.28E-21 | postive |
| NUBPL | AC093827.4 | 0.465709 | 1.24E-25 | postive |
| NUBPL | AC095055.1 | 0.531816 | 5.52E-36 | postive |
| NUBPL | AC096921.2 | 0.519002 | 8.5E-34 | postive |
| NUBPL | AC097376.3 | 0.447669 | 3.53E-23 | postive |
| NUBPL | AC097534.1 | 0.401665 | 1.51E-17 | postive |
| NUBPL | AC097639.1 | 0.497688 | 2.33E-30 | postive |
| NUBPL | AC103746.1 | 0.435685 | 1.26E-21 | postive |
| NUBPL | AC104088.3 | 0.404449 | 7.27E-18 | postive |
| NUBPL | AC104596.1 | 0.56716 | 1.57E-42 | postive |
| NUBPL | AC104819.3 | 0.409308 | 2E-18 | postive |
| NUBPL | AC107027.3 | 0.527132 | 3.57E-35 | postive |
| NUBPL | AC107068.1 | 0.491184 | 2.34E-29 | postive |
| NUBPL | AC108053.1 | 0.432283 | 3.37E-21 | postive |
| NUBPL | AC108693.2 | 0.422613 | 5.27E-20 | postive |
| NUBPL | AC109347.1 | 0.426067 | 1.99E-20 | postive |
| NUBPL | AC112220.2 | 0.611294 | 6.73E-52 | postive |
| NUBPL | AC120114.1 | 0.461677 | 4.52E-25 | postive |
| NUBPL | AC121338.2 | 0.49351 | 1.03E-29 | postive |
| NUBPL | AC124854.1 | 0.410928 | 1.3E-18 | postive |
| NUBPL | AC253536.3 | 0.415471 | 3.79E-19 | postive |
| NUBPL | ACVR2B-AS1 | 0.568196 | 9.84E-43 | postive |
| NUBPL | ADNP-AS1 | 0.418697 | 1.56E-19 | postive |
| NUBPL | AF111167.2 | 0.437911 | 6.54E-22 | postive |
| NUBPL | AF127577.4 | 0.507298 | 7.04E-32 | postive |
| NUBPL | AF241728.2 | 0.449529 | 2E-23 | postive |
| NUBPL | AL022069.1 | 0.402314 | 1.27E-17 | postive |
| NUBPL | AL023806.1 | 0.426317 | 1.86E-20 | postive |
| NUBPL | AL031778.1 | 0.41824 | 1.77E-19 | postive |
| NUBPL | AL035411.3 | 0.416843 | 2.6E-19 | postive |
| NUBPL | AL049779.4 | 0.402214 | 1.31E-17 | postive |
| NUBPL | AL049796.1 | 0.400644 | 1.97E-17 | postive |
| NUBPL | AL049840.3 | 0.434697 | 1.68E-21 | postive |
| NUBPL | AL049840.4 | 0.450823 | 1.35E-23 | postive |
| NUBPL | AL078581.2 | 0.564577 | 5.04E-42 | postive |
| NUBPL | AL078644.1 | 0.41669 | 2.72E-19 | postive |
| NUBPL | AL118506.1 | 0.430386 | 5.83E-21 | postive |
| NUBPL | AL121603.2 | 0.495497 | 5.11E-30 | postive |
| NUBPL | AL121820.1 | 0.411067 | 1.25E-18 | postive |
| NUBPL | AL132800.1 | 0.692623 | 1.15E-73 | postive |
| NUBPL | AL133227.1 | 0.430581 | 5.51E-21 | postive |
| NUBPL | AL135925.1 | 0.45749 | 1.7E-24 | postive |
| NUBPL | AL136169.1 | 0.402096 | 1.35E-17 | postive |
| NUBPL | AL137003.1 | 0.522291 | 2.38E-34 | postive |
| NUBPL | AL137779.2 | 0.529656 | 1.31E-35 | postive |
| NUBPL | AL139147.1 | 0.441071 | 2.57E-22 | postive |
| NUBPL | AL158212.3 | 0.44228 | 1.79E-22 | postive |
| NUBPL | AL161782.1 | 0.52786 | 2.67E-35 | postive |
| NUBPL | AL162171.1 | 0.590326 | 2.88E-47 | postive |
| NUBPL | AL162377.1 | 0.453928 | 5.17E-24 | postive |
| NUBPL | AL353748.3 | 0.500794 | 7.62E-31 | postive |
| NUBPL | AL353804.1 | 0.41554 | 3.72E-19 | postive |
| NUBPL | AL354811.1 | 0.420146 | 1.05E-19 | postive |
| NUBPL | AL355075.6 | 0.410619 | 1.41E-18 | postive |
| NUBPL | AL359317.1 | 0.462008 | 4.07E-25 | postive |
| NUBPL | AL359715.3 | 0.468244 | 5.46E-26 | postive |
| NUBPL | AL391834.1 | 0.408245 | 2.66E-18 | postive |
| NUBPL | AL450326.1 | 0.44273 | 1.57E-22 | postive |
| NUBPL | AL606834.1 | 0.476963 | 3.07E-27 | postive |
| NUBPL | AL732509.1 | 0.477668 | 2.43E-27 | postive |
| NUBPL | AP000759.1 | 0.416896 | 2.57E-19 | postive |
| NUBPL | AP000787.1 | 0.401978 | 1.39E-17 | postive |
| NUBPL | AP000866.1 | 0.502788 | 3.69E-31 | postive |
| NUBPL | AP001267.3 | 0.522198 | 2.47E-34 | postive |
| NUBPL | AP001318.2 | 0.450366 | 1.55E-23 | postive |
| NUBPL | AP001372.2 | 0.554832 | 3.71E-40 | postive |
| NUBPL | AP001625.2 | 0.408167 | 2.72E-18 | postive |
| NUBPL | AP001893.1 | 0.409706 | 1.8E-18 | postive |
| NUBPL | AP003721.3 | 0.429159 | 8.28E-21 | postive |
| NUBPL | ARHGAP31-AS1 | 0.484354 | 2.51E-28 | postive |
| NUBPL | ATXN1-AS1 | 0.404616 | 6.96E-18 | postive |
| NUBPL | BAALC-AS1 | 0.41002 | 1.66E-18 | postive |
| NUBPL | CCNT2-AS1 | 0.538324 | 3.93E-37 | postive |
| NUBPL | CHROMR | 0.400181 | 2.22E-17 | postive |
| NUBPL | CKMT2-AS1 | 0.495673 | 4.79E-30 | postive |
| NUBPL | COX10-AS1 | 0.488538 | 5.91E-29 | postive |
| NUBPL | CTBP1-DT | 0.541386 | 1.11E-37 | postive |
| NUBPL | DHRS4-AS1 | 0.626923 | 1.39E-55 | postive |
| NUBPL | DNAJC3-DT | 0.517799 | 1.35E-33 | postive |
| NUBPL | EDRF1-DT | 0.481872 | 5.86E-28 | postive |
| NUBPL | EIF2AK3-DT | 0.42181 | 6.59E-20 | postive |
| NUBPL | EIF3J-DT | 0.465772 | 1.22E-25 | postive |
| NUBPL | EMX2OS | 0.541495 | 1.06E-37 | postive |
| NUBPL | EPB41L4A-DT | 0.407351 | 3.38E-18 | postive |
| NUBPL | FAM111A-DT | 0.436233 | 1.07E-21 | postive |
| NUBPL | FAM160A1-DT | 0.442261 | 1.8E-22 | postive |
| NUBPL | FBXO30-DT | 0.529584 | 1.35E-35 | postive |
| NUBPL | FGD5-AS1 | 0.626713 | 1.56E-55 | postive |
| NUBPL | GAS5-AS1 | 0.471645 | 1.8E-26 | postive |
| NUBPL | GNG12-AS1 | 0.613172 | 2.49E-52 | postive |
| NUBPL | IQCH-AS1 | 0.425165 | 2.57E-20 | postive |
| NUBPL | ITGA9-AS1 | 0.407272 | 3.45E-18 | postive |
| NUBPL | KTN1-AS1 | 0.441245 | 2.44E-22 | postive |
| NUBPL | LINC00476 | 0.408118 | 2.75E-18 | postive |
| NUBPL | LINC00571 | 0.423488 | 4.12E-20 | postive |
| NUBPL | LINC00630 | 0.418876 | 1.49E-19 | postive |
| NUBPL | LINC00641 | 0.460906 | 5.78E-25 | postive |
| NUBPL | LINC00667 | 0.480181 | 1.04E-27 | postive |
| NUBPL | LINC00863 | 0.534323 | 2.01E-36 | postive |
| NUBPL | LINC00886 | 0.404311 | 7.54E-18 | postive |
| NUBPL | LINC01132 | 0.439239 | 4.42E-22 | postive |
| NUBPL | LINC01415 | 0.446148 | 5.6E-23 | postive |
| NUBPL | LINC01521 | 0.503913 | 2.45E-31 | postive |
| NUBPL | LINC01534 | 0.47283 | 1.21E-26 | postive |
| NUBPL | LINC01550 | 0.421438 | 7.31E-20 | postive |
| NUBPL | LINC01801 | 0.458766 | 1.14E-24 | postive |
| NUBPL | LINC02256 | 0.427004 | 1.53E-20 | postive |
| NUBPL | MAGI2-AS3 | 0.511509 | 1.47E-32 | postive |
| NUBPL | MALINC1 | 0.484358 | 2.51E-28 | postive |
| NUBPL | MCPH1-AS1 | 0.445606 | 6.6E-23 | postive |
| NUBPL | MIR4453HG | 0.480534 | 9.23E-28 | postive |
| NUBPL | MKLN1-AS | 0.441863 | 2.03E-22 | postive |
| NUBPL | MSC-AS1 | 0.413313 | 6.82E-19 | postive |
| NUBPL | NNT-AS1 | 0.622899 | 1.29E-54 | postive |
| NUBPL | NR2F1-AS1 | 0.453466 | 5.96E-24 | postive |
| NUBPL | NRSN2-AS1 | 0.460018 | 7.66E-25 | postive |
| NUBPL | NUTM2A-AS1 | 0.469852 | 3.23E-26 | postive |
| NUBPL | OTUD6B-AS1 | 0.677334 | 4.91E-69 | postive |
| NUBPL | OVCH1-AS1 | 0.436429 | 1.01E-21 | postive |
| NUBPL | PAXIP1-AS2 | 0.655934 | 5.22E-63 | postive |
| NUBPL | PLBD1-AS1 | 0.453085 | 6.71E-24 | postive |
| NUBPL | PPP1R12A-AS1 | 0.406276 | 4.49E-18 | postive |
| NUBPL | PPP3CB-AS1 | 0.468173 | 5.59E-26 | postive |
| NUBPL | PRKAR2A-AS1 | 0.441081 | 2.56E-22 | postive |
| NUBPL | PSMA3-AS1 | 0.405612 | 5.35E-18 | postive |
| NUBPL | PTOV1-AS1 | 0.44984 | 1.82E-23 | postive |
| NUBPL | RAP2C-AS1 | 0.732755 | 2.54E-87 | postive |
| NUBPL | RBM26-AS1 | 0.460095 | 7.47E-25 | postive |
| NUBPL | SBF2-AS1 | 0.425084 | 2.63E-20 | postive |
| NUBPL | SDCBP2-AS1 | 0.408079 | 2.78E-18 | postive |
| NUBPL | SEPTIN7-DT | 0.585157 | 3.54E-46 | postive |
| NUBPL | SETBP1-DT | 0.463841 | 2.26E-25 | postive |
| NUBPL | SGMS1-AS1 | 0.550109 | 2.83E-39 | postive |
| NUBPL | SLC25A21-AS1 | 0.4326 | 3.08E-21 | postive |
| NUBPL | SMARCA5-AS1 | 0.543349 | 4.91E-38 | postive |
| NUBPL | SMC5-AS1 | 0.440418 | 3.12E-22 | postive |
| NUBPL | SNHG14 | 0.454118 | 4.87E-24 | postive |
| NUBPL | SNHG16 | 0.412776 | 7.89E-19 | postive |
| NUBPL | SP2-AS1 | 0.471111 | 2.14E-26 | postive |
| NUBPL | ST7-AS1 | 0.442769 | 1.55E-22 | postive |
| NUBPL | STARD7-AS1 | 0.41628 | 3.04E-19 | postive |
| NUBPL | SUCLG2-AS1 | 0.602338 | 7.05E-50 | postive |
| NUBPL | TAPT1-AS1 | 0.430233 | 6.09E-21 | postive |
| NUBPL | TBC1D8-AS1 | 0.412793 | 7.85E-19 | postive |
| NUBPL | TMEM220-AS1 | 0.471669 | 1.78E-26 | postive |
| NUBPL | TMEM30A-DT | 0.467547 | 6.85E-26 | postive |
| NUBPL | TNFRSF10A-AS1 | 0.402344 | 1.26E-17 | postive |
| NUBPL | TRAM2-AS1 | 0.500208 | 9.42E-31 | postive |
| NUBPL | TRHDE-AS1 | 0.426039 | 2.01E-20 | postive |
| NUBPL | U91328.1 | 0.585005 | 3.81E-46 | postive |
| NUBPL | UBE2D3-AS1 | 0.414827 | 4.52E-19 | postive |
| NUBPL | UBL7-AS1 | 0.538496 | 3.66E-37 | postive |
| NUBPL | UGDH-AS1 | 0.64094 | 4.53E-59 | postive |
| NUBPL | USP27X-AS1 | 0.506784 | 8.51E-32 | postive |
| NUBPL | USP46-DT | 0.619172 | 9.92E-54 | postive |
| NUBPL | UXT-AS1 | 0.474946 | 6.02E-27 | postive |
| NUBPL | WASL-DT | 0.516111 | 2.57E-33 | postive |
| NUBPL | WDFY3-AS2 | 0.50548 | 1.38E-31 | postive |
| NUBPL | ZKSCAN7-AS1 | 0.516422 | 2.28E-33 | postive |
| NUBPL | ZNF197-AS1 | 0.416241 | 3.07E-19 | postive |
| NUBPL | ZNF22-AS1 | 0.431025 | 4.85E-21 | postive |
| NUBPL | ZNF503-AS1 | 0.42542 | 2.39E-20 | postive |
| NUBPL | ZNF667-AS1 | 0.470989 | 2.23E-26 | postive |
| NUBPL | ZNF674-AS1 | 0.403851 | 8.51E-18 | postive |
| OXSM | AC005076.1 | 0.406026 | 4.8E-18 | postive |
| OXSM | AC005082.1 | 0.404905 | 6.45E-18 | postive |
| OXSM | AC005696.1 | 0.491311 | 2.24E-29 | postive |
| OXSM | AC006213.1 | 0.431282 | 4.5E-21 | postive |
| OXSM | AC008763.1 | 0.404583 | 7.02E-18 | postive |
| OXSM | AC012313.1 | 0.426714 | 1.66E-20 | postive |
| OXSM | AC012640.2 | 0.404816 | 6.6E-18 | postive |
| OXSM | AC024575.1 | 0.502149 | 4.66E-31 | postive |
| OXSM | AC068338.2 | 0.446641 | 4.82E-23 | postive |
| OXSM | AC079848.1 | 0.453974 | 5.09E-24 | postive |
| OXSM | AC092295.2 | 0.444321 | 9.72E-23 | postive |
| OXSM | AC093599.2 | 0.401171 | 1.71E-17 | postive |
| OXSM | AC097359.2 | 0.426879 | 1.58E-20 | postive |
| OXSM | AC103563.7 | 0.490691 | 2.79E-29 | postive |
| OXSM | AC112220.2 | 0.543927 | 3.86E-38 | postive |
| OXSM | AC116036.2 | 0.400668 | 1.95E-17 | postive |
| OXSM | AC139768.1 | 0.452582 | 7.84E-24 | postive |
| OXSM | ACVR2B-AS1 | 0.447946 | 3.25E-23 | postive |
| OXSM | AL031123.4 | 0.430672 | 5.37E-21 | postive |
| OXSM | AL050341.2 | 0.428891 | 8.94E-21 | postive |
| OXSM | AL118558.3 | 0.400937 | 1.82E-17 | postive |
| OXSM | AL132800.1 | 0.42535 | 2.44E-20 | postive |
| OXSM | AL161665.2 | 0.43163 | 4.07E-21 | postive |
| OXSM | AL162171.1 | 0.511168 | 1.67E-32 | postive |
| OXSM | AL162377.1 | 0.481885 | 5.83E-28 | postive |
| OXSM | AL353572.4 | 0.423679 | 3.91E-20 | postive |
| OXSM | AL355001.2 | 0.451641 | 1.05E-23 | postive |
| OXSM | AP000894.4 | 0.407859 | 2.95E-18 | postive |
| OXSM | AP003068.2 | 0.406228 | 4.55E-18 | postive |
| OXSM | ARIH2OS | 0.517298 | 1.63E-33 | postive |
| OXSM | EIF3J-DT | 0.506116 | 1.09E-31 | postive |
| OXSM | ERVE-1 | 0.405127 | 6.08E-18 | postive |
| OXSM | FGD5-AS1 | 0.531639 | 5.92E-36 | postive |
| OXSM | FLJ37453 | 0.404954 | 6.37E-18 | postive |
| OXSM | GAS6-DT | 0.412738 | 7.97E-19 | postive |
| OXSM | HOXB-AS3 | 0.453436 | 6.02E-24 | postive |
| OXSM | ILF3-DT | 0.435997 | 1.15E-21 | postive |
| OXSM | LINC00886 | 0.436796 | 9.07E-22 | postive |
| OXSM | LINC01003 | 0.492565 | 1.44E-29 | postive |
| OXSM | NNT-AS1 | 0.49585 | 4.5E-30 | postive |
| OXSM | NRSN2-AS1 | 0.4425 | 1.68E-22 | postive |
| OXSM | OTUD6B-AS1 | 0.443138 | 1.39E-22 | postive |
| OXSM | POLR2J4 | 0.440445 | 3.09E-22 | postive |
| OXSM | PRKAR2A-AS1 | 0.492714 | 1.37E-29 | postive |
| OXSM | RNASEH1-AS1 | 0.512662 | 9.5E-33 | postive |
| OXSM | SBF2-AS1 | 0.465489 | 1.33E-25 | postive |
| OXSM | SCAMP1-AS1 | 0.51478 | 4.27E-33 | postive |
| OXSM | TMEM220-AS1 | 0.404828 | 6.58E-18 | postive |
| OXSM | U91328.1 | 0.40691 | 3.8E-18 | postive |
| OXSM | UBAC2-AS1 | 0.44165 | 2.16E-22 | postive |
| OXSM | USP27X-AS1 | 0.420934 | 8.41E-20 | postive |
| OXSM | WASL-DT | 0.433281 | 2.53E-21 | postive |
| OXSM | ZBED5-AS1 | 0.406907 | 3.8E-18 | postive |
| OXSM | ZNF503-AS2 | 0.499587 | 1.18E-30 | postive |
| OXSM | ZNF561-AS1 | 0.456566 | 2.27E-24 | postive |
| OXSM | ZNF582-AS1 | 0.482753 | 4.34E-28 | postive |
| OXSM | ZNF793-AS1 | 0.480406 | 9.64E-28 | postive |
| RPN1 | AC092368.3 | 0.437978 | 6.41E-22 | postive |
| RPN1 | AC107027.3 | 0.473954 | 8.37E-27 | postive |
| RPN1 | ZBTB11-AS1 | 0.535951 | 1.04E-36 | postive |
| SLC3A2 | AC097359.2 | 0.424585 | 3.03E-20 | postive |
| SLC3A2 | AC144652.1 | 0.445408 | 7E-23 | postive |
| SLC3A2 | AL118558.3 | 0.458009 | 1.44E-24 | postive |
| SLC3A2 | AP000757.1 | 0.400299 | 2.15E-17 | postive |
| SLC3A2 | AP003068.2 | 0.417507 | 2.17E-19 | postive |
| SLC3A2 | ATP6V0E2-AS1 | 0.403792 | 8.64E-18 | postive |
| SLC3A2 | GTSE1-DT | 0.410522 | 1.45E-18 | postive |
| SLC3A2 | LINC01003 | 0.408332 | 2.6E-18 | postive |
| SLC3A2 | SLC25A5-AS1 | 0.417252 | 2.33E-19 | postive |
| SLC7A11 | LINC01270 | 0.470864 | 2.32E-26 | postive |
| SLC7A11 | LINC02693 | 0.422091 | 6.1E-20 | postive |
