# Supplementary material for: Development of a novel disulfidptosis-related lncRNA signature for prognostic and immune response prediction in clear cell renal cell carcinoma
Source: Sci Rep. 2024 Jan 5;14:624. doi: 10.1038/s41598-024-51197-2 (PMC10770353; doi:10.1038/s41598-024-51197-2)
Supplement: Supplementary file 2 — Supplementary Table S2. [file 41598_2024_51197_MOESM2_ESM.docx]

**Supplementary Table S2** Univariate cox regression analysis of training set.

| id | HR | HR.95L | HR.95H | *P*-value |
| --- | --- | --- | --- | --- |
| AP000696.2 | 2.323221 | 1.18867796 | 4.540638345 | 0.013682369 |
| AC006116.9 | 0.100412 | 0.02648917 | 0.380630097 | 0.000723041 |
| AC015802.5 | 2.475655 | 1.50783925 | 4.064668036 | 0.00033923 |
| AC084024.4 | 2.575231 | 1.61291906 | 4.111683916 | 7.42E-05 |
| AL353748.3 | 0.521148 | 0.33991895 | 0.798998512 | 0.002797321 |
| MAP4K3-DT | 0.374273 | 0.1884163 | 0.743460693 | 0.005008142 |
| AC099791.2 | 2.145264 | 1.55631538 | 2.957086603 | 3.14E-06 |
| AP001542.3 | 0.667037 | 0.55152033 | 0.80674957 | 3.00E-05 |
| TRAM2-AS1 | 0.517459 | 0.34914265 | 0.766917287 | 0.001030977 |
| NRAV | 0.684181 | 0.48118638 | 0.97281208 | 0.034561554 |
| AC073073.2 | 0.5247 | 0.32387369 | 0.850054493 | 0.008795341 |
| AC015922.2 | 0.769632 | 0.64133421 | 0.923595039 | 0.004889775 |
| AC004112.1 | 0.559366 | 0.37319438 | 0.838412533 | 0.004900417 |
| LINC01132 | 0.574868 | 0.38050385 | 0.868514813 | 0.008550003 |
| AC108693.2 | 0.381453 | 0.21266115 | 0.684218685 | 0.001225401 |
| CTBP1-DT | 0.650511 | 0.46134747 | 0.917236365 | 0.014177251 |
| ZNF503-AS2 | 0.578407 | 0.38717556 | 0.864089799 | 0.007512231 |
| GAS5-AS1 | 0.487282 | 0.30329854 | 0.782871651 | 0.002959874 |
| AL592295.6 | 0.670784 | 0.47860441 | 0.940130333 | 0.020426974 |
| AC091982.3 | 0.695314 | 0.50688416 | 0.953791906 | 0.024238778 |
| AC009962.1 | 0.419883 | 0.18465495 | 0.95476232 | 0.038413695 |
| USP46-DT | 0.679363 | 0.48653566 | 0.948611747 | 0.023226608 |
| ZKSCAN7-AS1 | 0.471262 | 0.29099262 | 0.763206989 | 0.002224299 |
| OVCH1-AS1 | 0.230353 | 0.10581664 | 0.501458041 | 0.000216418 |
| PAXIP1-AS2 | 0.592596 | 0.42657612 | 0.823229585 | 0.00180995 |
| AP003068.2 | 0.636245 | 0.47384568 | 0.854304 | 0.002636407 |
| FZD4-DT | 0.516851 | 0.32950592 | 0.81071256 | 0.004058221 |
| SNHG29 | 0.634175 | 0.50555068 | 0.795525039 | 8.22E-05 |
| LINC00571 | 0.471867 | 0.2898632 | 0.768149358 | 0.00252023 |
| AC103563.7 | 0.748903 | 0.56823273 | 0.987017587 | 0.040098425 |
| PPP1R12A-AS1 | 0.594741 | 0.36103878 | 0.979719781 | 0.041308866 |
| AC092329.4 | 0.526778 | 0.28088984 | 0.98791538 | 0.045731835 |
| SNHG8 | 0.532639 | 0.39419768 | 0.719700527 | 4.10E-05 |
| LINCMD1 | 0.561125 | 0.41498788 | 0.758723666 | 0.000174195 |
| AL158206.1 | 0.729479 | 0.54092611 | 0.983757246 | 0.038706541 |
| AL132800.1 | 0.529534 | 0.3792148 | 0.739439579 | 0.000190042 |
| NNT-AS1 | 0.642018 | 0.48003268 | 0.858665665 | 0.002816442 |
| AF111167.2 | 0.577587 | 0.37958898 | 0.878863181 | 0.010381057 |
| AC007637.1 | 0.62291 | 0.46081318 | 0.842026117 | 0.002083526 |
| AC026748.7 | 0.722281 | 0.54163839 | 0.96316929 | 0.026725143 |
| AC021037.1 | 0.26069 | 0.09752571 | 0.696834371 | 0.007362151 |
| AL121832.2 | 1.389924 | 1.03537848 | 1.865878034 | 0.028425726 |
| AC093510.1 | 0.254667 | 0.11100664 | 0.584244756 | 0.001244382 |
| EIF3J-DT | 0.621946 | 0.45001425 | 0.859566059 | 0.004019971 |
| AL162377.1 | 0.401814 | 0.24862562 | 0.64938822 | 0.000197138 |
| AC097639.1 | 0.44852 | 0.29877472 | 0.673316111 | 0.000109642 |
| CASC2 | 0.475665 | 0.24812341 | 0.911872818 | 0.025233711 |
| HAGLR | 0.791695 | 0.66260199 | 0.945939388 | 0.010113774 |
| U91328.1 | 0.583453 | 0.42716712 | 0.796918856 | 0.000706756 |
| ENTPD3-AS1 | 0.522643 | 0.28485286 | 0.958937756 | 0.036138404 |
| AC005670.3 | 0.614902 | 0.43971759 | 0.859880064 | 0.004478641 |
| AL137779.2 | 0.647826 | 0.43003358 | 0.975921765 | 0.037843634 |
| AC093827.4 | 0.597255 | 0.38171501 | 0.934501687 | 0.024036341 |
| AC104109.2 | 0.43952 | 0.26097728 | 0.740210102 | 0.001994246 |
| SNHG9 | 1.426042 | 1.11673962 | 1.821011754 | 0.004439756 |
| AC103746.1 | 0.606504 | 0.40553283 | 0.907072487 | 0.014896481 |
| AL035071.1 | 2.092008 | 1.45442942 | 3.009081088 | 6.90E-05 |
| AP001505.1 | 1.93499 | 1.39701187 | 2.680138991 | 7.14E-05 |
| ZNF561-AS1 | 0.54117 | 0.29296274 | 0.99966752 | 0.049875988 |
| AC018521.6 | 0.670133 | 0.4754665 | 0.944500765 | 0.022250422 |
| TRHDE-AS1 | 0.693915 | 0.52830912 | 0.911433257 | 0.008625155 |
| GNG12-AS1 | 0.353157 | 0.20343858 | 0.613059159 | 0.000216705 |
| AC017100.1 | 0.525887 | 0.36062882 | 0.766875134 | 0.000840711 |
| CCDC183-AS1 | 0.635912 | 0.46894261 | 0.862331113 | 0.003578739 |
| SNHG25 | 1.400893 | 1.14571487 | 1.712905037 | 0.001016733 |
| CARD8-AS1 | 0.718405 | 0.51618449 | 0.999847048 | 0.049894083 |
| AP003721.4 | 0.477246 | 0.28984992 | 0.785797584 | 0.003644348 |
| AL121944.1 | 0.253835 | 0.11500708 | 0.560245468 | 0.000687998 |
| AC068492.1 | 1.662272 | 1.21286897 | 2.27819091 | 0.001577631 |
| AC064807.1 | 0.607087 | 0.42184135 | 0.873681277 | 0.007209675 |
| AC011912.1 | 0.482903 | 0.24970059 | 0.933898824 | 0.030526789 |
| AC131009.3 | 1.54206 | 1.15941149 | 2.050996724 | 0.002916244 |
| LINC01671 | 0.803403 | 0.708774 | 0.910666835 | 0.000618217 |
| AC068338.2 | 0.441859 | 0.25018219 | 0.780388473 | 0.004886967 |
| EMX2OS | 0.728408 | 0.63926254 | 0.829983901 | 1.96E-06 |
| WASL-DT | 0.589788 | 0.40994993 | 0.84851937 | 0.004439797 |
| AC021218.1 | 0.836261 | 0.72391754 | 0.966039881 | 0.015125444 |
| AC018809.2 | 0.484173 | 0.28440273 | 0.824267297 | 0.007542455 |
| DHRS4-AS1 | 0.685663 | 0.48546847 | 0.968411639 | 0.032179698 |
| FAM111A-DT | 0.65872 | 0.43981082 | 0.986587129 | 0.042817791 |
| AC139768.1 | 0.598721 | 0.39834184 | 0.899896132 | 0.013613857 |
| AC009554.2 | 1.45911 | 1.09195432 | 1.94971588 | 0.010625032 |
| AC034139.1 | 0.52334 | 0.31212351 | 0.877488527 | 0.014065745 |
| LINC01550 | 0.622597 | 0.47674896 | 0.813063603 | 0.000502155 |
| AC021087.1 | 0.652885 | 0.45456397 | 0.937732556 | 0.020999742 |
| AC087623.2 | 1.988335 | 1.21678956 | 3.249105177 | 0.00608651 |
| OTUD6B-AS1 | 0.543393 | 0.38914974 | 0.758771545 | 0.000342892 |
| SMARCA5-AS1 | 0.416828 | 0.26447508 | 0.656946141 | 0.000163176 |
| LINC01534 | 0.686393 | 0.47573896 | 0.990322222 | 0.044224226 |
| AC112220.2 | 0.430651 | 0.27791315 | 0.667332247 | 0.000163301 |
| AC104088.3 | 0.174278 | 0.07841107 | 0.387352668 | 1.81E-05 |
| AC003086.1 | 0.613458 | 0.4247507 | 0.886004255 | 0.009180144 |
| ZNF503-AS1 | 0.360305 | 0.22773472 | 0.570047763 | 1.29E-05 |
| UGDH-AS1 | 0.647672 | 0.43946375 | 0.954524732 | 0.02815134 |
| AC106791.1 | 0.392054 | 0.23791834 | 0.646045121 | 0.000238466 |
| WDFY3-AS2 | 0.591599 | 0.4474853 | 0.782124803 | 0.000228578 |
| AC096921.2 | 0.697699 | 0.51517078 | 0.944899424 | 0.020006348 |
| SGMS1-AS1 | 0.526899 | 0.32678913 | 0.849545377 | 0.008564379 |
| LINC01852 | 0.528538 | 0.35334389 | 0.790595556 | 0.001911501 |
| TMEM220-AS1 | 0.425759 | 0.2238331 | 0.80984846 | 0.009244658 |
| AC092296.1 | 0.42435 | 0.2556713 | 0.704313112 | 0.000913324 |
| AP001267.3 | 0.332089 | 0.17775344 | 0.620427718 | 0.000546468 |
| AL035411.3 | 0.308811 | 0.1103507 | 0.864191314 | 0.025223508 |
| AC026992.2 | 0.556577 | 0.39738616 | 0.779537536 | 0.000652277 |
| EPB41L4A-DT | 0.45462 | 0.3448321 | 0.599362625 | 2.27E-08 |
| EDRF1-DT | 0.228118 | 0.08490695 | 0.612881272 | 0.003379996 |
| AC008966.1 | 0.549362 | 0.30686643 | 0.983486233 | 0.043799205 |
| AL021707.6 | 1.419822 | 1.14052841 | 1.767508984 | 0.001709488 |
| AC107027.3 | 0.578822 | 0.39414588 | 0.850026229 | 0.005291587 |
| AC079848.1 | 0.578126 | 0.39107953 | 0.854633736 | 0.006003149 |
| AL031733.2 | 2.633518 | 1.59309506 | 4.353423846 | 0.000159497 |
| AL161729.3 | 2.130459 | 1.21124432 | 3.747266965 | 0.008661073 |
| AC121338.2 | 0.471282 | 0.34046195 | 0.652367786 | 5.77E-06 |
| AC004554.1 | 0.650829 | 0.44191299 | 0.958511739 | 0.029668341 |
| GAS6-DT | 0.647233 | 0.471916 | 0.88768005 | 0.006951349 |
| AL359715.3 | 0.524963 | 0.32180043 | 0.856388545 | 0.00985597 |
| AC009486.1 | 0.545023 | 0.3473031 | 0.855305165 | 0.008296488 |
| AL513165.1 | 0.758822 | 0.58016487 | 0.992494542 | 0.043908069 |
| GATA3-AS1 | 1.723834 | 1.00129913 | 2.967747592 | 0.049454995 |
| SPINT1-AS1 | 0.63583 | 0.48010242 | 0.842070059 | 0.001582055 |
| PLBD1-AS1 | 0.462726 | 0.31332708 | 0.683359441 | 0.000107099 |
| AC108673.3 | 1.910258 | 1.41260878 | 2.583224288 | 2.63E-05 |
| AL158212.3 | 0.677979 | 0.47472319 | 0.968259312 | 0.032569132 |
| LINC00886 | 0.542683 | 0.37423943 | 0.786941446 | 0.001265825 |
| LINC01415 | 0.409899 | 0.23213005 | 0.723804818 | 0.002111201 |
| LINC01270 | 2.794806 | 1.49758944 | 5.215676096 | 0.001243733 |
| AP001207.3 | 3.563879 | 1.05943738 | 11.98866187 | 0.040048494 |
| AC008555.1 | 0.673614 | 0.53878944 | 0.842176509 | 0.000525561 |
| AC018752.1 | 0.668603 | 0.54327783 | 0.822839554 | 0.000144009 |
| CYTOR | 1.886155 | 1.24316399 | 2.861715843 | 0.002851671 |
| LINC01801 | 0.586833 | 0.43933958 | 0.783843314 | 0.000307387 |
| AC006213.1 | 0.201421 | 0.06259454 | 0.64814784 | 0.007205817 |
| AC005034.5 | 0.627086 | 0.43619483 | 0.901516562 | 0.011743494 |
| ITGA9-AS1 | 0.300382 | 0.11442992 | 0.78851422 | 0.014585747 |
| AC008937.3 | 0.412938 | 0.21371188 | 0.79788672 | 0.008492622 |
| AL078581.2 | 0.4667 | 0.34047512 | 0.63972161 | 2.17E-06 |
| AC008543.1 | 0.447891 | 0.21915011 | 0.915382789 | 0.027637588 |
| RAP2C-AS1 | 0.467138 | 0.25941147 | 0.8412041 | 0.011207827 |
| AL161782.1 | 0.394767 | 0.28158027 | 0.553452127 | 6.98E-08 |
| AL162171.1 | 0.620125 | 0.44622163 | 0.861801419 | 0.004431118 |
| AC104596.1 | 0.526949 | 0.32002887 | 0.867658155 | 0.011806182 |
| AC018647.2 | 0.62977 | 0.44214681 | 0.897009225 | 0.01040041 |
| AC073254.1 | 0.45986 | 0.26616829 | 0.794501732 | 0.005360438 |
| FGD5-AS1 | 0.641666 | 0.46507212 | 0.885315717 | 0.006898719 |
| ACVR2B-AS1 | 0.672734 | 0.47869978 | 0.945415963 | 0.022414444 |
| AL450326.1 | 0.600219 | 0.44652735 | 0.806809249 | 0.000718603 |
| EPB41L4A-AS1 | 0.693168 | 0.54149728 | 0.887321772 | 0.003627797 |
| LINC02027 | 0.616976 | 0.47217353 | 0.806184736 | 0.000402251 |
| SEPTIN7-DT | 0.52243 | 0.34380075 | 0.793868755 | 0.002356127 |
| AC068338.3 | 0.542952 | 0.31125731 | 0.947114988 | 0.031447598 |
| BNC2-AS1 | 1.419437 | 1.14605378 | 1.758034578 | 0.001332463 |
| AC124854.1 | 0.77165 | 0.68516647 | 0.869050798 | 1.92E-05 |
| AC017099.2 | 0.497535 | 0.32874085 | 0.752998625 | 0.000960886 |
| USP27X-AS1 | 0.463255 | 0.3156229 | 0.679942106 | 8.49E-05 |
| SP2-AS1 | 0.59201 | 0.3696012 | 0.948254446 | 0.029182202 |
| AP001372.2 | 0.478226 | 0.32820564 | 0.696819981 | 0.000122679 |
| RBM26-AS1 | 0.568486 | 0.34386353 | 0.939839182 | 0.027675192 |
| AL035587.2 | 2.400123 | 1.49732503 | 3.847254416 | 0.000276044 |
| AL606489.1 | 0.770118 | 0.6001782 | 0.988175793 | 0.040026829 |
| LINC01963 | 0.580469 | 0.43262469 | 0.77883719 | 0.000287308 |
| NR2F1-AS1 | 0.614855 | 0.38290263 | 0.987319525 | 0.044138182 |
| AC012313.5 | 0.581483 | 0.40452459 | 0.835851844 | 0.00340671 |
| SNAI3-AS1 | 0.481187 | 0.24292296 | 0.953143934 | 0.035942862 |
| AC095055.1 | 0.39891 | 0.25762834 | 0.617669686 | 3.79E-05 |
| AC046143.2 | 1.882939 | 1.34816948 | 2.629833168 | 0.000205134 |
| AP003721.3 | 0.464006 | 0.3297034 | 0.653016823 | 1.06E-05 |
| ZNF582-AS1 | 0.561176 | 0.38362878 | 0.820894242 | 0.00291131 |
| MRPS30-DT | 0.617252 | 0.41562635 | 0.916689176 | 0.016800214 |
| LINC02188 | 0.795343 | 0.66089249 | 0.957145833 | 0.015370198 |
| SUCLG2-AS1 | 0.352284 | 0.19544414 | 0.634983281 | 0.000518899 |
| AL359878.2 | 2.714062 | 1.72295218 | 4.275297677 | 1.66E-05 |
| ARHGAP31-AS1 | 0.593205 | 0.42128576 | 0.835281437 | 0.002782862 |
| CAMTA1-DT | 1.878851 | 1.29407013 | 2.727889765 | 0.000916306 |
| AL078644.1 | 0.560659 | 0.37054413 | 0.84831704 | 0.006172374 |
| CCNT2-AS1 | 0.589914 | 0.3499908 | 0.994307837 | 0.047547033 |
| SBF2-AS1 | 0.486881 | 0.32495726 | 0.72949128 | 0.000485021 |
| AC007743.1 | 0.565024 | 0.42875731 | 0.74459808 | 5.03E-05 |
| AC108053.1 | 0.440537 | 0.28823689 | 0.673309757 | 0.000152168 |
| POLR2J4 | 0.458811 | 0.28851395 | 0.72962788 | 0.000995574 |
| SNHG14 | 0.616778 | 0.40507494 | 0.939122738 | 0.024274094 |
| AP000759.1 | 0.687291 | 0.49217949 | 0.959749032 | 0.027728234 |

**Supplementary Table S2**

| Disulfidptosis | lncRNA | | | |
| --- | --- | --- | --- | --- |
| GYS1 | LINC01522 | PINK1-AS | AC114803.1 | LINC02188 |
| GYS1 | LINC01426 |  |  |  |
| LRPPRC | UXT-AS1 | NNT-AS1 | LINC01671 | AP001267.3 |
|  | AC084024.4 | AC026748.7 | EMX2OS | EIF2AK3-DT |
|  | AL353748.3 | AC021037.1 | WASL-DT | EDRF1-DT |
|  | MAP4K3-DT | AL590428.1 | AC021218.1 | AC008966.1 |
|  | NIFK-AS1 | STK4-AS1 | DHRS4-AS1 | AC107027.3 |
|  | AC120114.1 | EIF3J-DT | FAM111A-DT | AC079848.1 |
|  | AP001542.3 | LINC00863 | AC011477.2 | AC121338.2 |
|  | TRAM2-AS1 | AC091978.1 | AC034139.1 | AC004554.1 |
|  | NRAV | HAGLR | LINC01550 | AC108673.3 |
|  | AC073073.2 | U91328.1 | OTUD6B-AS1 | SNHG16 |
|  | AC015922.2 | AC005670.3 | AL732509.1 | AC008555.1 |
|  | AC004112.1 | AC007365.1 | SMARCA5-AS1 | AC018752.1 |
|  | CTBP1-DT | AL035071.1 | LINC01534 | LINC01801 |
|  | AL592295.6 | AC093297.2 | AC112220.2 | AC006213.1 |
|  | AC091982.3 | GNG12-AS1 | AC003086.1 | AC005034.5 |
|  | USP46-DT | AP003721.4 | UGDH-AS1 | AL078581.2 |
|  | ZNF22-AS1 | AC064807.1 | WDFY3-AS2 | BAIAP2-DT |
|  | HMGN3-AS1 | AC011912.1 | SGMS1-AS1 | RAP2C-AS1 |
|  | PAXIP1-AS2 | LINC01521 | AC002401.2 | AL161782.1 |
|  | FZD4-DT | MKLN1-AS | AL132800.1 | AL162171.1 |
|  | SNHG29 | AC092667.1 | EPB41L4A-AS1 | AC104596.1 |
|  | UBL7-AS1 | FGD5-AS1 | SEPTIN7-DT | AC018647.2 |
|  | ATXN1-AS1 | ACVR2B-AS1 | TNFRSF10A-AS1 | FAM160A1-DT |
|  | SNHG8 | AL450326.1 | LINCMD1 | HCG15 |
|  | NRSN2-AS1 | RBM26-AS1 | SUCLG2-AS1 | AC073254.1 |
|  | AC124854.1 | AL606489.1 | AL078644.1 | SBF2-AS1 |
|  | USP27X-AS1 | LINC01963 | AL022069.3 | AC007743.1 |
|  | SP2-AS1 | AC095055.1 | MALINC1 | AC005332.6 |
|  | AC016727.1 | TBC1D8-AS1 | CCNT2-AS1 | AC006994.1 |
|  | AP001372.2 | AP003721.3 | MRPS30-DT | AL137003.1 |
|  | AP000759.1 |  |  |  |
| NCKAP1 | NIFK-AS1 | LINC01963 | AL161782.1 | AC107027.3 |
| NCKAP1 | AC120114.1 | AC095055.1 | AC104596.1 | AP001318.2 |
| NCKAP1 | AP001542.3 | AP003721.3 | AC009318.3 | AC004554.1 |
| NCKAP1 | TRAM2-AS1 | MRPS30-DT | AC018647.2 | NUTM2A-AS1 |
| NCKAP1 | NRAV | SUCLG2-AS1 | AC073254.1 | AL121603.2 |
| NCKAP1 | AC073073.2 | AL078644.1 | FGD5-AS1 | AC007485.1 |
| NCKAP1 | AC015922.2 | AL022069.3 | AL450326.1 | AC108673.3 |
| NCKAP1 | AC004112.1 | MALINC1 | TNFRSF10A-AS1 | SNHG16 |
| NCKAP1 | CTBP1-DT | CCNT2-AS1 | LINC02027 | AC015922.3 |
| NCKAP1 | AL592295.6 | AC007743.1 | SEPTIN7-DT | LINC01415 |
| NCKAP1 | AC009962.1 | AC005332.6 | NRSN2-AS1 | SETBP1-DT |
| NCKAP1 | USP46-DT | LINC00667 | AC017099.2 | AC108463.2 |
| NCKAP1 | ZNF22-AS1 | AL137003.1 | USP27X-AS1 | AC008555.1 |
| NCKAP1 | HMGN3-AS1 | AP000759.1 | SP2-AS1 | AC018752.1 |
| NCKAP1 | PAXIP1-AS2 | AC068870.2 | AC016727.1 | AC090198.1 |
| NCKAP1 | FZD4-DT | SMARCA5-AS1 | AP001372.2 | LINC01801 |
| NCKAP1 | UBL7-AS1 | AC112220.2 | RBM26-AS1 | AC006213.1 |
| NCKAP1 | MSC-AS1 | AC003086.1 | AL606489.1 | AC005034.5 |
| NCKAP1 | AL158206.1 | AC103591.4 | AC018645.3 | AL078581.2 |
| NCKAP1 | AL132800.1 | UGDH-AS1 | AC005670.3 | BAIAP2-DT |
| NCKAP1 | WAC-AS1 | WDFY3-AS2 | AC007405.3 | RAP2C-AS1 |
| NCKAP1 | NNT-AS1 | AC096921.2 | DNAJC3-DT | AF127577.4 |
| NCKAP1 | AC021037.1 | SGMS1-AS1 | AC007365.1 | EMX2OS |
| NCKAP1 | AL590428.1 | AC002401.2 | GNG12-AS1 | AC012306.2 |
| NCKAP1 | STK4-AS1 | EIF2AK3-DT | AC083799.1 | WASL-DT |
| NCKAP1 | EIF3J-DT | AC244517.7 | CARD8-AS1 | CLCA4-AS1 |
| NCKAP1 | LINC00863 | AC091563.1 | AL135925.1 | AC009318.2 |
| NCKAP1 | AC097639.1 | AC011912.1 | AC008124.1 | DHRS4-AS1 |
| NCKAP1 | CASC2 | LINC01521 | AF241728.2 | FAM111A-DT |
| NCKAP1 | HAGLR | MKLN1-AS | AC064807.1 | AC011477.2 |
| NCKAP1 | U91328.1 | LINC01671 | AC108047.1 | AC034139.1 |
| NCKAP1 | AL732509.1 | OTUD6B-AS1 |  |  |
| NDUFA11 | AP000696.2 | Z97192.2 | AC046143.2 | SLC25A5-AS1 |
|  | AC015802.5 | ANK3-DT | AL359878.2 | LINC01521 |
|  | LINC01983 | AC034102.3 | CAMTA1-DT | AC068338.2 |
|  | AC099791.2 | AC004080.4 | AC012065.2 | EMX2OS |
|  | AC009309.1 | AC087379.2 | FARSA-AS1 | AL023806.1 |
|  | LINC01976 | PRR34-AS1 | AC133552.5 | WASL-DT |
|  | AC006538.1 | AC009554.2 | LINC01023 | DHRS4-AS1 |
|  | AP007216.2 | AC087623.2 | Z97653.1 | AC011477.2 |
|  | AL391883.1 | SEMA3B-AS1 | SUCLA2-AS1 | OTUD6B-AS1 |
|  | AC092306.1 | AL354953.1 | CDC37L1-DT | SMARCA5-AS1 |
|  | RNF207-AS1 | AL023803.1 | AL353748.3 | AC112220.2 |
|  | HEIH | LAMA5-AS1 | MAP4K3-DT | AC106791.1 |
|  | AC008622.2 | AP003307.1 | AP001542.3 | WDFY3-AS2 |
|  | AC040169.1 | AC092119.3 | NRAV | SGMS1-AS1 |
|  | PRDM16-DT | AL021707.6 | AC015922.2 | AC002401.2 |
|  | AC008897.3 | AL031733.2 | AC004112.1 | LINC01852 |
|  | AP003032.1 | AL161729.3 | AC108693.2 | AC092296.1 |
|  | AL121832.2 | AC103724.4 | GAS5-AS1 | AL035411.3 |
|  | RAB11B-AS1 | HSD11B1-AS1 | AL592295.6 | EIF2AK3-DT |
|  | TBX2-AS1 | AL513165.1 | USP46-DT | AC026992.2 |
|  | AC010913.1 | GATA3-AS1 | PCAT7 | AC107027.3 |
|  | AC104316.2 | AC130371.2 | HMGN3-AS1 | AC079848.1 |
|  | FOXC2-AS1 | AC067852.2 | PAXIP1-AS2 | AC004554.1 |
|  | ENTPD3-AS1 | SPINT1-AS1 | LINC00571 | PLBD1-AS1 |
|  | SNHG9 | SNHG19 | NNT-AS1 | AC005034.5 |
|  | AC008915.2 | AP001207.3 | AF111167.2 | AL449106.1 |
|  | AP001505.1 | CYTOR | AC007637.1 | AL078581.2 |
|  | AC018529.1 | AC026803.2 | AC021037.1 | RAP2C-AS1 |
|  | SNHG25 | AC023509.3 | AL162377.1 | AL161782.1 |
|  | AL121899.1 | AC099518.6 | LINC00863 | ZNF710-AS1 |
|  | AL121944.1 | AL355353.1 | U91328.1 | AC018647.2 |
|  | AC068492.1 | AP001363.2 | AC005670.3 | AC073254.1 |
|  | AC131009.3 | LIPE-AS1 | DNAJC3-DT | AC097359.2 |
|  | RPARP-AS1 | AC068338.3 | AC104109.2 | FGD5-AS1 |
|  | AL391261.2 | BNC2-AS1 | AC017100.1 | AL450326.1 |
|  | ZSCAN16-AS1 | CH17-340M24.3 | CCDC183-AS1 | LINC02027 |
|  | AC009090.1 | BX649632.1 | AC008124.1 | AC092295.2 |
|  | AC005498.2 | AL035587.2 | AC064807.1 | AL606489.1 |
|  | AC012313.5 | SNAI3-AS1 | AL359704.2 | AL512603.2 |
|  | AC026691.1 | AL138966.2 | TAF1A-AS1 | AP003721.3 |
|  | PPIC-AS1 | SUCLG2-AS1 | AL136040.1 | PTOV1-AS1 |
| NUBPL | UXT-AS1 | AC107068.1 | PSMA3-AS1 | COX10-AS1 |
|  | AC006116.9 | AL049840.3 | LINC01521 | SMC5-AS1 |
|  | MAGI2-AS3 | LINC00641 | ST7-AS1 | WDFY3-AS2 |
|  | AL353748.3 | AL132800.1 | AC087071.2 | AC026471.1 |
|  | AC120114.1 | NNT-AS1 | MKLN1-AS | AP000866.1 |
|  | AC011477.1 | LINC00630 | CKMT2-AS1 | AC096921.2 |
|  | TRAM2-AS1 | AF111167.2 | AC253536.3 | SGMS1-AS1 |
|  | AC073073.2 | AC007637.1 | AC068620.1 | AL121820.1 |
|  | AC015922.2 | AC093510.1 | AF127577.4 | TMEM220-AS1 |
|  | AC004112.1 | AL022069.1 | EMX2OS | AP001267.3 |
|  | LINC01132 | EIF3J-DT | AP001625.2 | AL035411.3 |
|  | AC108693.2 | AL162377.1 | AL355075.6 | EIF2AK3-DT |
|  | AL118506.1 | AL139147.1 | AC010615.2 | EPB41L4A-DT |
|  | CTBP1-DT | LINC00863 | AL023806.1 | AL049840.4 |
|  | AC026979.4 | AL133227.1 | WASL-DT | EDRF1-DT |
|  | SLC25A21-AS1 | AC097639.1 | AP000787.1 | AC107027.3 |
|  | GAS5-AS1 | AC093227.3 | AC018809.2 | AP001318.2 |
|  | AC009962.1 | AC036214.2 | AC009318.2 | AC121338.2 |
|  | MIR4453HG | U91328.1 | DHRS4-AS1 | AC004554.1 |
|  | USP46-DT | AC005670.3 | FAM111A-DT | AL359715.3 |
|  | ZKSCAN7-AS1 | AL137779.2 | KTN1-AS1 | NUTM2A-AS1 |
|  | ZNF22-AS1 | DNAJC3-DT | AC011477.2 | AC009486.1 |
|  | OVCH1-AS1 | AC093827.4 | AC034139.1 | AL121603.2 |
|  | PAXIP1-AS2 | AC103746.1 | IQCH-AS1 | AC007066.2 |
|  | AL359317.1 | AL049796.1 | LINC01550 | AC012313.1 |
|  | UBL7-AS1 | AC007365.1 | AC021087.1 | AC109347.1 |
|  | LINC00571 | AL606834.1 | AL354811.1 | PLBD1-AS1 |
|  | PPP1R12A-AS1 | BAALC-AS1 | AC092802.1 | LINC00476 |
|  | ATXN1-AS1 | AC104819.3 | OTUD6B-AS1 | AC020915.2 |
|  | TAPT1-AS1 | AC093297.2 | AL732509.1 | AL136169.1 |
|  | MSC-AS1 | AC018521.6 | SMARCA5-AS1 | AC022211.2 |
|  | AC092329.4 | TRHDE-AS1 | LINC01534 | AL158212.3 |
|  | AC046134.2 | GNG12-AS1 | AC112220.2 | SNHG16 |
|  | AC008494.2 | AC008537.2 | AC104088.3 | AC097376.3 |
|  | AC079142.1 | AL049779.4 | AC078883.1 | LINC00886 |
|  | AC008555.1 | AL135925.1 | ZNF503-AS1 | STARD7-AS1 |
|  | AC018752.1 | AC008124.1 | PRKAR2A-AS1 | LINC01415 |
|  | AC090198.1 | AF241728.2 | UGDH-AS1 | LINC02256 |
|  | LINC01801 | AC064807.1 | AC108053.1 | SETBP1-DT |
|  | AC006213.1 | AC011912.1 | AC005332.6 | AP001893.1 |
|  | AC005034.5 | SP2-AS1 | AC006994.1 | ARHGAP31-AS1 |
|  | AL031778.1 | AC016727.1 | LINC00667 | AL078644.1 |
|  | ITGA9-AS1 | AP001372.2 | AL137003.1 | PTOV1-AS1 |
|  | MCPH1-AS1 | RBM26-AS1 | SNHG14 | MALINC1 |
|  | AC008937.3 | PPP3CB-AS1 | AC004918.3 | AL353804.1 |
|  | AL078581.2 | UBE2D3-AS1 | TMEM30A-DT | CCNT2-AS1 |
|  | ZNF197-AS1 | ZNF674-AS1 | AP000759.1 | CHROMR |
|  | AC008543.1 | NR2F1-AS1 | TNFRSF10A-AS1 | SBF2-AS1 |
|  | RAP2C-AS1 | AC012313.5 | SEPTIN7-DT | AC007743.1 |
|  | AL391834.1 | AC020779.2 | AC005498.3 | USP27X-AS1 |
|  | AL161782.1 | FBXO30-DT | NRSN2-AS1 | FAM160A1-DT |
|  | AL162171.1 | AC095055.1 | AC092295.2 | FGD5-AS1 |
|  | AC104596.1 | TBC1D8-AS1 | AC124854.1 | ACVR2B-AS1 |
|  | AC006213.4 | AC097534.1 | SDCBP2-AS1 | AL450326.1 |
|  | AC018647.2 | AP003721.3 | AC017099.2 | SUCLG2-AS1 |
|  | ZNF667-AS1 | ADNP-AS1 |  |  |
| OXSM | ZNF503-AS2 | EIF3J-DT | AC139768.1 | PRKAR2A-AS1 |
|  | AC116036.2 | AL162377.1 | OTUD6B-AS1 | AL355001.2 |
|  | AP003068.2 | ZBED5-AS1 | AC112220.2 | TMEM220-AS1 |
|  | AL353572.4 | U91328.1 | LINC01003 | AC005082.1 |
|  | AC103563.7 | ZNF561-AS1 | AC005076.1 | AC079848.1 |
|  | AL161665.2 | AL050341.2 | AC006213.1 | SCAMP1-AS1 |
|  | ILF3-DT | ZNF793-AS1 | AL162171.1 | FLJ37453 |
|  | AL132800.1 | AC068338.2 | AC097359.2 | AC005696.1 |
|  | NNT-AS1 | HOXB-AS3 | FGD5-AS1 | AL031123.4 |
|  | AC093599.2 | WASL-DT | ACVR2B-AS1 | GAS6-DT |
|  | ERVE-1 | AL118558.3 | NRSN2-AS1 | AC012313.1 |
|  | USP27X-AS1 | UBAC2-AS1 | AC092295.2 | LINC00886 |
|  | AC008763.1 | ZNF582-AS1 | AC024575.1 | SBF2-AS1 |
|  | ARIH2OS | AP000894.4 | AC012640.2 | RNASEH1-AS1 |
|  | POLR2J4 |  |  |  |
| RPN1 | AC092368.3 | AC107027.3 | ZBTB11-AS1 |  |
| SLC3A2 | ATP6V0E2-AS1 | LINC01003 | AC144652.1 | SLC25A5-AS1 |
|  | AP000757.1 | AC097359.2 | GTSE1-DT | AL118558.3 |
|  | AP003068.2 |  |  |  |
| SLC7A11 | LINC02693 | LINC01270 |  |  |
