# Supplementary figures and images for: Development of a novel disulfidptosis-related lncRNA signature for prognostic and immune response prediction in clear cell renal cell carcinoma
Source: Sci Rep. 2024 Jan 5;14:624. doi: 10.1038/s41598-024-51197-2 (PMC10770353; doi:10.1038/s41598-024-51197-2)

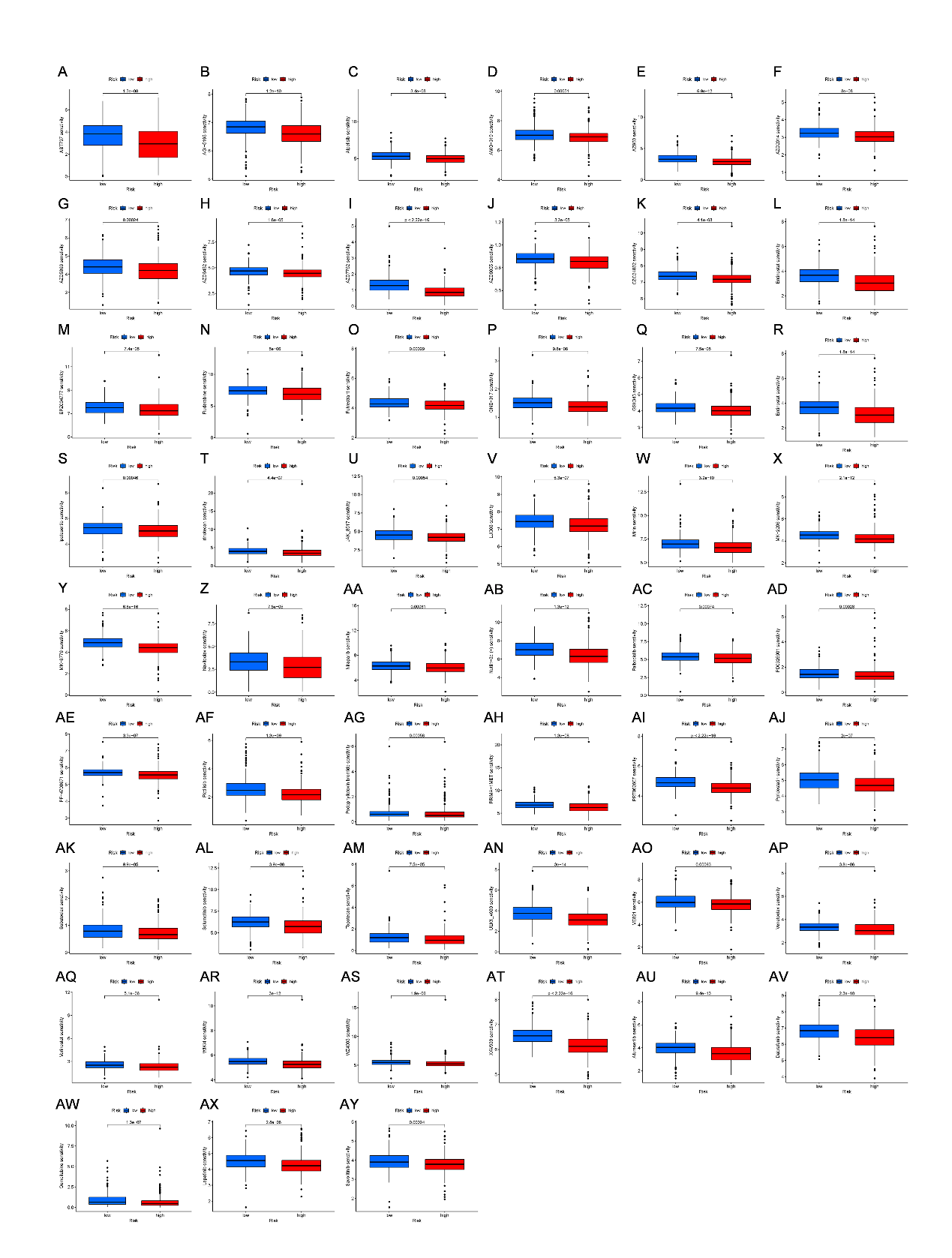


**Figure S1** Drug sensitivity. **(A-AY)** Drugs sensitive to high-risk groups**.**

Supplement: Supplementary file 3 — Supplementary Figure S1. [file 41598_2024_51197_MOESM3_ESM.docx]

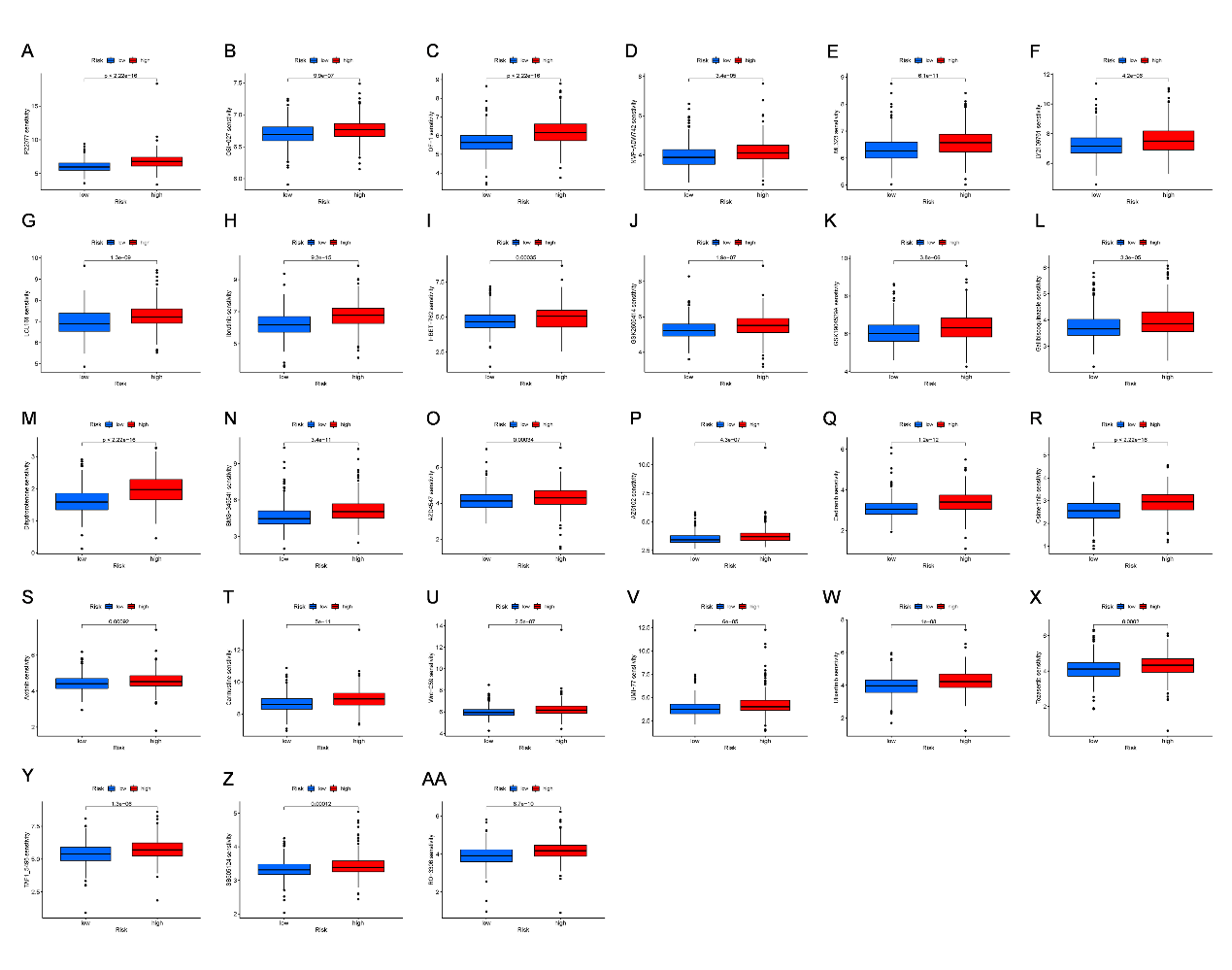


**Figure S2** Drug sensitivity. **(A-AA)** Drugs sensitive to low-risk groups**.**

Supplement: Supplementary file 4 — Supplementary Figure S2. [file 41598_2024_51197_MOESM4_ESM.docx]
